# Engineering tumor-specific catalytic nanosystem for NIR-II photothermal-augmented and synergistic starvation/chemodynamic nanotherapy

**DOI:** 10.1186/s40824-022-00317-y

**Published:** 2022-11-26

**Authors:** Shuixiu Zhou, Jiahuan Xu, Yanfei Dai, Yan Wei, Liang Chen, Wei Feng, Yu Chen, Xuejun Ni

**Affiliations:** 1 Department of Medical Ultrasound, Affiliated Hospital of Nantong University, Nantong, 226001 People’s Republic of China; 2 Radiology Department, Branch of Affiliated Hospital of Nantong University, Nantong, 226001 People’s Republic of China; 3 Materdicine Lab, School of Life Sciences, Shanghai University, Shanghai, 200444 People’s Republic of China

**Keywords:** Polyoxometalate, Photothermal therapy, Chemodynamic therapy, Glucose oxidase, Starvation therapy

## Abstract

**Background:**

As an emerging therapeutic modality, chemodynamic therapy (CDT), converting hydrogen peroxide (H_2_O_2_) into highly toxic reactive oxygen species (ROS), has been developed for tumor-specific therapy. However, the deficiency of endogenous H_2_O_2_ and high concentration of glutathione (GSH) in the tumor microenvironment (TME) weaken the CDT-based tumor-therapeutic efficacy. Herein, a photothermal-enhanced tumor-specific cascade catalytic nanosystem has been constructed on the basis of glucose oxidase (GOD)-functionalized molybdenum (Mo)-based polyoxometalate (POM) nanoclusters, termed as GOD@POMs.

**Methods:**

GOD@POMs were synthesized by a facile one-pot procedure and covalently conjugation. Then, its structure was characterized by scanning electron microscope (SEM), transmission electron microscope (TEM), Fourier transform infrared (FTIR) spectroscopy and X-ray photoelectron spectroscopy (XPS). In addition, ultraviolet-visible-near-infrared (UV-vis-NIR) absorption spectrum and infrared thermal camera were applied to evaluate the catalytic and photothermal performance, respectively. Moreover, to confirm the therapeutic effects in vitro, cell counting kit-8 (CCK-8) assay, live/dead staining and ROS staining were performed. Furthermore, the biosafety of GOD@POMs was investigated via blood routine, blood biochemistry and hematoxylin and eosin (H&E) staining in Kunming mice. Besides, the C6 glioma tumor-bearing mice were constructed to evaluate its anti-tumor effects in vivo and its photoacoustic (PA) imaging capability. Notably, RNA sequencing, H&E, TdT-mediated dUTP nick end labeling (TUNEL) and Ki-67 staining were also conducted to disclose its underlying anti-tumor mechanism.

**Results:**

In this multifunctional nanosystem, GOD can effectively catalyze the oxidation of intratumoral glucose into gluconic acid and H_2_O_2_, achieving the cancer starvation therapy. Meanwhile, the generated gluconic acid decreases the pH in TME resulting in POM aggregation, which enables PA imaging-guided tumor-specific photothermal therapy (PTT), especially in the second near-infrared (NIR-II) biological window. Importantly, the Mo (VI) sites on POM can be reduced to Mo (V) active sites in accompany with GSH depletion, and then the post-produced Mo (V) transforms in situ overproduced H_2_O_2_ into singlet oxygen (^1^O_2_) via Russell mechanism, achieving self-enhanced CDT. Moreover, the PTT-triggered local tumor temperature elevation augments the synergistic nanocatalytic-therapeutic efficacy.

**Conclusions:**

Consequently, the integration of GOD-induced starvation therapy, H_2_O_2_ self-supply/GSH-depletion enhanced Mo-based CDT, and POM aggregation-mediated PTT endow the GOD@POMs with remarkable synergistic anticancer outcomes with neglectable adverse effects.

**Supplementary Information:**

The online version contains supplementary material available at 10.1186/s40824-022-00317-y.

## Introduction

Currently, cancer is a kind of serious disease threatening public health. According to statistics, estimated 608,570 Americans died from cancer in 2021, corresponding to more than 1600 deaths per day [1]. The unmet clinical challenges encountered in cancer therapeutics, including drug resistance, high grade normal tissue toxicity and difficulty in deep tumor treatment, have led to the alternative approaches for development. Chemodynamic therapy (CDT), based on Fenton/Fenton-like reactions, introducing specific valence ions including iron (Fe), manganese (Mn), cobalt (Co), titanium (Ti), cerium (Ce) and copper (Cu) to react with endogenous hydrogen peroxide (H_2_O_2_) for generating more highly toxic and active reactive oxygen species (ROS) such as hydroxyl radicals (•OH) to kill tumor cells, is regarded as a promising tumor-therapeutic modality with high potential in clinical transformation [2–11]. However, a large amount of antioxidants mainly involving glutathione (GSH, up to 10 mM) exist in tumor microenvironment (TME), which plays a vital role in counteracting ROS-mediated oxidative damage during the treatment, thus the therapeutic effect of CDT is substantially neutralized [12, 13]. Furthermore, although tumor cells are gifted with relatively high H_2_O_2_ level (100 × 10^− 6^ to 1 × 10^− 3^ M) compared with normal cells, the endogenous H_2_O_2_ in the tumor region is generally insufficient to generate abundant •OH. Additionally, the occurrence and progress of Fenton/Fenton-like reaction demands rigorous reaction condition such as acidic environment pH ranging from 2 to 4.5 so that the mildly acidic character pH between 6.5 and 6.9 in TME cannot effectively initiate the reaction at a high degree [14, 15]. Taken together, effective TME regulation including eliminating intratumoral GSH, elevating endogenous H_2_O_2_ level and increasing acidity is clinically urgent to develop efficient strategies to improve the catalytic performance for CDT [16, 17].

On the basis of Warburg effect, the proliferating cancer cells consume much more nutrients such as glucose than normal cells to meet their own energy demand for cell survival, growth and differentiation. Glucose oxidase (GOD)-mediated metabolism reacts with glucose in TME to produce H_2_O_2_ and gluconic acid, which blocks the tumorous energy supply by consuming glucose, thereby starving tumor cells death [18–20]. Furthermore, the generated H_2_O_2_ provides abundant reactants for CDT, meanwhile accompanying with generated gluconic acid for inducing more acidic environment, consequently improving the therapeutic efficiency of CDT [21, 22]. Moreover, it has been proven that when the temperature achieves at 50 °C, the catalytic activity of GOD reaches its maximum. Photothermal therapy (PTT) employing photothermal agents (PTAs) converts the near-infrared (NIR) light energy into heat energy in target region for ablating tumors [23]. Notably, compared with the traditional first near-infrared (NIR-I) biological window 650–950 nm, the second near-infrared (NIR-II) biological window 1000–1350 nm possesses several remarkable advantages, such as deeper penetration of biological tissues and less tissue scattering and absorption. Although great efforts have been devoted to exploring and developing PTAs including plasmonic metals (e.g., palladium nanosheets, gold nanostructures) [24, 25], transition metal dichalcogenides and oxides [26, 27], two-dimensional (2D) metal carbides and nitrides (MXenes, e.g., Ti_3_C_2_, Nb_2_C, Mo_2_C, V_2_C, Ta_3_C_4_ and W_1.33_C) [28–31], 2D monoelement materials (Xenes, e.g., silicene, germanene, phosphorene, arsenene, antimonene, bismuthene) [32, 33], and conjugated polymers [34, 35], some critical issues are still required to be addressed. The non-specificity, reduced therapeutic efficacy, unclear long-term biological consequences and off-target heating-induced noncancerous regions damage are Gordian knots for current PTAs. Therefore, improving the accumulation of PTAs in tumor tissues based on TME is one of the most desirable strategies for efficient tumor treatment.

**Scheme 1:**
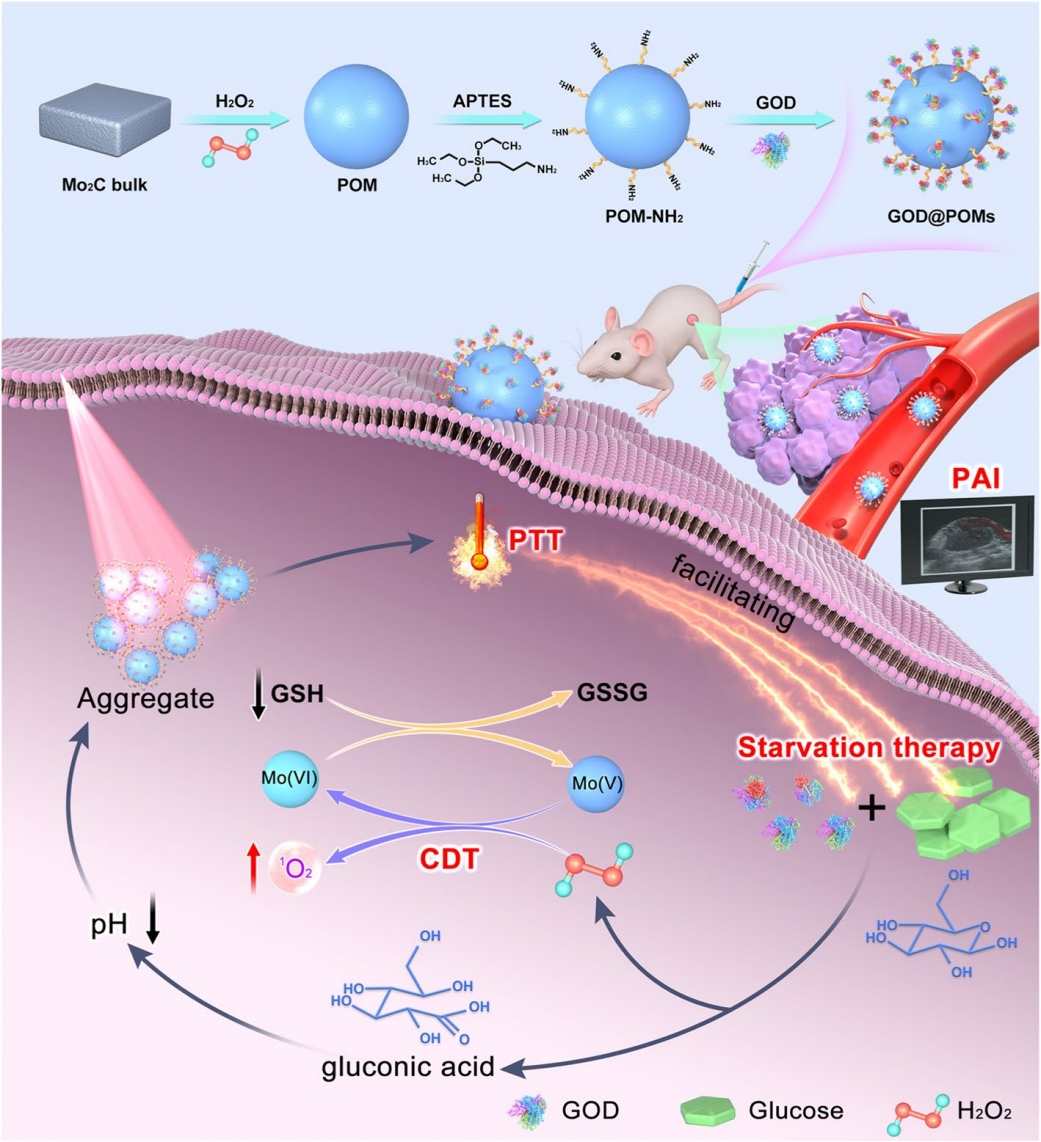
Schematic illustration of the synthetic process of GOD@POMs and the corresponding therapeutic mechanism on tumor treatment. In this nanosystem, GOD-induced starvation therapy, H_2_O_2_ self-supply/GSH-depletion enhanced Mo-based CDT, and POM aggregation-mediated PTT, which not only improves the catalytic efficiency of GOD but also promotes the Mo-mediated CDT process

In this work, we have designed and engineered a kind of intelligent molybdenum (Mo)-based polyoxometalate (POM) nanoclusters modified with GOD (GOD@POMs) as a multifunctional therapeutic catalytic nanosystem based on the strategy of photonic hyperthermia-reinforced and specific TME-triggered cascaded nanocatalytic cancer treatment (Scheme 1). When these multifunctional GOD@POMs enter the tumor region, GOD serves as the start point to deplete glucose for blocking the nutrients/energy supply to tumor cells. Furthermore, the post-produced H_2_O_2_ could oxidize the Mo (V) into Mo (VI) that results in the efficient formation of cytotoxic singlet oxygen (^1^O_2_) on the basis of Russell mechanism, which is unlike traditional Fenton-like agents (Fe, Cu and Mn) that generate noxious hydroxyl radicals (•OH), and the Mo (VI) could consume GSH to form Mo(V). Meanwhile, compared to other PTAs, pH-responsive Mo-based POM may aggregate from small nanoparticle to big ones in mildly acidic tumors, enabling the tumor-specific targeting therapy and enhancing the photothermal conversion. Thus, the decreased pH in TME resulting from the formed gluconic acid promotes the self-assembly and self-adaptive photothermal conversion of Mo-based POM, which could serve as a desirable NIR-II PTAs. Under the NIR-II laser irradiation, the light-to-heat conversion capacity of POM elevates the local temperature, achieving efficient PTT, which not only improves the catalytic efficiency of GOD but also promotes the ROS generation during the Mo-mediated CDT process. This rationally engineered multifunctional nanosystem provides a distinct paradigm of collaborative treatment strategy to achieve highly efficient tumor treatment with desirable clinical translation prospects.

## Materials and methods

### Materials and reagents

Molybdenum carbide (Mo_2_C), 3-aminopropyltriethoxysilane (APTES), glucose oxidase (GOD), N-(3-Dim-ethylaminopropyl)-N^′^-ethylcarbodiimide hydrochloride (EDC), N-hydroxyl succinimide (NHS), hydrogen peroxide (H_2_O_2_), hydrochloric acid (HCl), 1, 3-diphenylisobenzofuran (DPBF), 2,7-dichlorofluorescein diacetate (DCFH-DA) and glucose were obtained from Sigma-Aldrich Trading Co., Ltd. (Shanghai, China). Cell counting kit-8 (CCK-8), calcein-AM and propidium iodide (PI) were purchased from Beyotime Biotechnology (Shanghai, China). Dulbecco’s modified Eagle medium (DMEM), fetal bovine serum (FBS), penicillin-streptomycin solution, phosphate buffer saline (PBS), and trypsin were purchased from Gibco Trading Co., Ltd. (Shanghai, China). All chemicals were used as received unless otherwise stated.

### Synthesis of polyoxometalate (POM)

The POM was synthesized from bulk Mo_2_C powders via one-pot oxidation reaction. 2 g Mo_2_C powder was dispersed in 15 mL deionized (DI) water with vigorous stirring. Then, 2 mL H_2_O_2_ was added drop by drop and the reaction was kept overnight. When the reaction was finished, Mo_2_C residue was removed by centrifugation (3000 rpm). After lyophilization, deep blue colored powder was obtained as Mo-based POM.

### Synthesis of polyoxometalate-amino (POM-NH_2_)

1.8 mg/mL POM aqueous solution was slowly added in 0.37 mL APTES. After the solution became clear, its pH was adjusted to 1.5 with 1 M HCl solution. The solution was then vigorously stirred for 24 h, and the precipitate was obtained by 10,000 rpm centrifugation. Finally, the precipitate was collected and washed with DI water, and dispersed in 2.5 mL DI water for further use.

### Synthesis of GOD@POMs

38 mg EDC, 57 mg NHS and 0.5 mg GOD were dissolved in 6 mL DI water followed by adding 1 mL POM-NH_2_ dispersion. The system was stirred for 8 h at room temperature to produce GOD@POMs. After centrifugation, the precipitate was collected and washed with DI water.

### Characterization

Morphologies were characterized using a SU8010 field emission scanning electron microscope (SEM, Hitachi, Japan) and transmission electron microscope (TEM, JEOL JEM-2100plus). Dynamic particle size was measured on a Zetasizer Nanoseries (Nano ZS90, Malvern Instrument Ltd.). Ultraviolet-visible-near-infrared (UV–vis–NIR) absorption spectrum was recorded using a Shimadzu UV3600 UV–vis–NIR scanning spectrometer (Shimadzu Scientific Instruments, Japan). Fourier transform infrared (FTIR) spectroscopy were collected with a Thermo Scienific™ Nicolet™ iS5 FTIR analyzer. GOD encapsulation was determined by Netzsch TG 209 F3 Tarsus. X-ray photoelectron spectroscopy (XPS) measurements were carried out on the Thermal Scientific™ K-Alpha™.

### Solution stabilities of GOD@POMs

GOD@POMs (Mo concentration: 1000 μmol/L) were dispersed in H_2_O and PBS respectively. After that, we recorded its absorption by UV-vis-NIR and took digital photos at 24 and 72 h respectively.

### Measurement of pH changes

After adding 1, 2 or 3 mg/mL glucose into GOD@POMs aqueous solution with same Mo concentration (1000 μmol/L), pH of each system was measured at an interval of 30 s, and the whole reaction process was kept for 300 s.

### Quantitative analysis of the ^1^O_2_ generation

DPBF was applied as a probe to detect the generation of ^1^O_2_ in GOD@POMs dispersion with existence of H_2_O_2_ and glucose. Firstly, GOD@POMs aqueous solution (Mo concentration: 1000 μmol/L) mixed with DPBF (1 mg/mL, dissolved in DMF), and then the test started when adding H_2_O_2_ (10 mM) or glucose (1 mg/mL) into the mixed solution. For the reaction between GOD@POMs and H_2_O_2_, the absorbance of DPBF at 420 nm was recorded every 2 min using UV–vis–NIR spectra. For the reaction between GOD@POMs and glucose, the absorbance of DPBF at 420 nm was recorded every 5 min.

### Photothermal performance of GOD@POMs

UV absorption of GOD@POMs with different Mo concentration (0, 250, 500, 750, 1000 and 1500 μmol/L) was detected. To record temperature-changes curves, GOD@POMs aqueous solutions with different Mo concentration (0, 250, 500, 750 and 1000 μmol/L) were adopted under NIR-II laser irradiation at different power densities (0.5, 0.8, 1.0 and 1.5 W/cm^2^) for 10 min. Subsequently, in order to evaluate the photothermal stability of GOD@POMs, temperature changes of GOD@POMs solution (Mo concentration: 1000 μmol/L) through five laser on/off cycles (1 W/cm^2^) were recorded.

### Cell culture

The mouse fibroblast cell line (L929 cells) is applied for cytotoxicity assessments. The C6 rat glioma cell line is used as a cell model for neurological tumor. Both the L929 mouse fibroblasts cell line and C6 rat glioma cell line was obtained from the Cell Bank of Shanghai Institutes for Biological Sciences, Chinese Academy of Sciences (Shanghai, China). All cells were regularly maintained in complete DMEM medium supplemented 10% FBS, 100 UmL^− 1^ penicillin and 100 UmL^− 1^ streptomycin and kept at 37 °C in a humidified atmosphere of 5% CO_2_ and 95% air. The medium was changed every two days, and the cells were routinely passaged by using 0.25% trypsin solution before approaching 80% confluence.

### Cell endocytosis of GOD@POMs

To confirm the endocytosis of GOD@POMs, fluorescein isothiocyanate (FITC) was applied to label GOD@POMs. C6 cells were seeded in confocal dishes and then incubated with FITC-labelled GOD@POMs. After co-incubation for 0 and 8 h, confocal laser scanning microscopy (CLSM) was used to detect cell endocytosis.

### In vitro cytotoxicity of GOD@POMs

To assess the cytotoxicity of GOD@POMs, L929 cells were seeded into a 96-well plate with the density of 1 × 10^4^ cells per well and then cultured at 37 °C for 24 h. Then, GOD@POMs dispersion were added into the wells and co-incubated for another 24 h. At the end of co-incubation, 100 μL 10-fold diluted CCK-8 were added into each well. After co-incubation for another 4 h, the absorbance of CCK-8 was monitored by a micro-plate reader at the wavelength of 450 nm.

### In vitro anti-tumor assessment

To verify the effects of reaction between GOD and glucose, glucose (25, 50, 100, 125 and 250 μg/mL) were reacted with GOD@POMs or POMs (Mo concentration: 1000 μmol/L) and then the standard CCK-8 assay was performed to access the cell viability. Besides, to test the photothermal ablation performance, C6 cells were seeded into a 96-well plate with the density of 1 × 10^4^ cells per well and then cultured overnight. Then, the C6 cells were incubated GOD@POMs or POMs with different Mo concentration (0, 250, 500, 750, 1000 and 1500 μmol/L). After 4 h incubation, the cells were exposed to the 1064 nm laser irradiation (1 W/cm^2^, 5 min). After that, the cell viabilities were measured by CCK-8 assay. To compare the therapeutic effects of different treatments: (I) Control; (II) Laser; (III) GOD@POMs; (IV) GOD@POMs + Laser; (V) GOD@POMs + Glucose; (VI) GOD@POMs + Glucose + Laser, the Mo concentration of GOD@POMs was 1000 μmol/L and the concentration of glucose was 1 mg/mL and the condition of 1064 nm laser treatment was 1 W/cm^2^ for 5 min. Finally, the cell survival rate of C6 cell was tested by the typical CCK-8 assay.

### Live-dead cell staining assay

To further prove the anti-tumor effects of GOD@POMs, the C6 cells were treated with six different protocols: (I) Control; (II) Laser; (III) GOD@POMs; (IV) GOD@POMs + Laser; (V) GOD@POMs + Glucose; (VI) GOD@POMs + Laser + Glucose. The Mo concentration of GOD@POMs was 1000 μmol/L and the concentration of glucose was 1 mg/mL. The condition for 1064 nm laser treatment was 1 W/cm^2^, 5 min. After diverse treatments, cells were double stained with calcein-AM (green color, live cells) and PI (red color, dead cells) and then observed by CLSM.

### Cell apoptosis analysis by flow cytometry

C6 cells were seeded into 6-well plates after different treatments, and cells were collected and stained with Annexin V-FITC and PI. In the end, the fluorescence intensity of cells was quantitatively detected by flow cytometry.

### Intracellular ^1^O_2_ generation

The ROS probe, DCFH-DA, was applied to test the intracellular generation of ROS. C6 cells were seeded into confocal dish at a density of 10^5^ cells per dish and incubated for 24 h. Then, the cells were treated as follows: (I) Control; (II) Laser; (III) GOD@POMs; (IV) GOD@POMs + Laser; (V) GOD@POMs + Glucose. The Mo concentration of GOD@POMs was 1000 μmol/L and the concentration of glucose was 1 mg/mL. The condition of 1064 nm laser treatment was  irradiated at the power density of 1 W/cm^2 ^for 5 min. After that, DCFH-DA was added into above treated cells. After 30 min incubation, the cells were washed three times by PBS. Finally, the intracellular ROS generation was determined by CLSM. Flow cytometry was also used to quantitatively detect ROS generation. C6 cells were seeded into 6-well plates, and then the DCFH-DA was added and co-incubated for 30 min. Finally, cells were treated with previous manners and then collected to detect the ROS by flow cytometry.

### Animals and treatment

Six-week-old healthy female Kunming mice and thirty BALB/C nude mice with 4–6 weeks were purchased from Slac Laboratory Animal Co., Ltd. (Shanghai, China). The animals were housed in stainless steel and ventilated cages under the standard conditions (temperature: 25 ± 2 °C, relative humidity: 60 ± 10%, and light: 12 h light/dark cycle) for seven days prior to treatment. All animal experiments were performed with the approval of the ethics by the Ethics Committee of Shanghai University (License number: ECSHU-2021-029).

### Photoacoustic (PA) imaging

Different Mo concentrations of GOD@POMs (250, 500, 750, 1000 and 1500 μmol/L) dissolved in purified water were performed with excitation wavelength of 808 nm and 1200 nm. For PA imaging in vivo, the C6 tumor bearing mice were intravenously injected with GOD@POMs (10 mg/kg). After the injection, the signal was recorded with excitation wavelength at 1200 nm.

### In vivo IR-thermal imaging

To verify the NIR-II laser-triggered tumor hyperthermia, C6 tumor bearing mice were intravenously injected with GOD@POMs (10 mg/kg). At 6 h post-injection, the tumors were irradiated with 1064 nm laser (1 W/cm^2^, 10 min). The photothermal heating curves and photothermal heating images of tumors at 0, 2, 4, 6, 8 and 10 min were recorded.

### In vivo toxicity assessment of GOD@POMs

Ten Kunming mice were assigned to two groups, which were intravenously injected with GOD@POMs (10 mg/kg), and mice injected with PBS were set as the control group. The body weight of the mice was measured every other day. The mice were anesthetized and dissected in 28 days of post-injection. The blood was taken out for blood routine and blood biochemical analysis. The major organs (heart, liver, spleen, lung and kidney) were dissected, fixed in a 10% formalin solution and stained with hematoxylin and eosin (H&E) for histological analysis.

### In vivo biodistribution

A C6 tumor-bearing model was established by subcutaneous inoculation of C6 cells (1 × 10^6^) on the right leg of BALB/C nude mice. To assess the in vivo biodistribution, Cyanine 5.5 (Cy5.5) was initially used to label GOD@POMs and then the in vivo fluorescence distribution at different time intervals were monitored by using in vivo imaging system (IVIS). After that, the mice were euthanized followed by collecting the organs including heart, liver, spleen, lung, kidney and tumors. The ex vivo fluorescent images were acquired.

### In vivo tumor therapy

Thirty C6 tumor bearing BALB/C nude mice were divided into six groups (*n* = 5 in each group): (I) Control; (II) Laser; (III) GOD; (IV) POMs; (V) GOD@POMs; (VI) GOD@POMs + Laser. For control group, the tumor-bearing mice were merely injected with PBS. For GOD and POMs group, the mice were injected with only GOD or only POMs. For GOD@POMs and GOD@POMs + Laser group, the mice were injected with GOD@POMs (10 mg/kg). Further, Laser and GOD@POMs + Laser groups were irradiated with 1064 nm laser at the power density of 1 W/cm^2 ^for 10 min at 6 h post-injection, and the tumor temperature was monitored by IR camera. The tumor volume and body weight were recorded every other day. After 15 days of treatment, all mice were sacrificed, and the main organ tissues of each group of mice were dissected for H&E histological analysis, TdT-mediated dUTP nickend labeling (TUNEL) and Ki-67 antibody staining to observe cell apoptosis and cell proliferation. Tumor volume was calculated by following equation: V = length × wide^2^ × 0.5, where length and wide denote the maximum length and maximum width of the tumor of the mice.

## Results

### Synthesis and characterizations of GOD@POMs

**Fig. 1:**
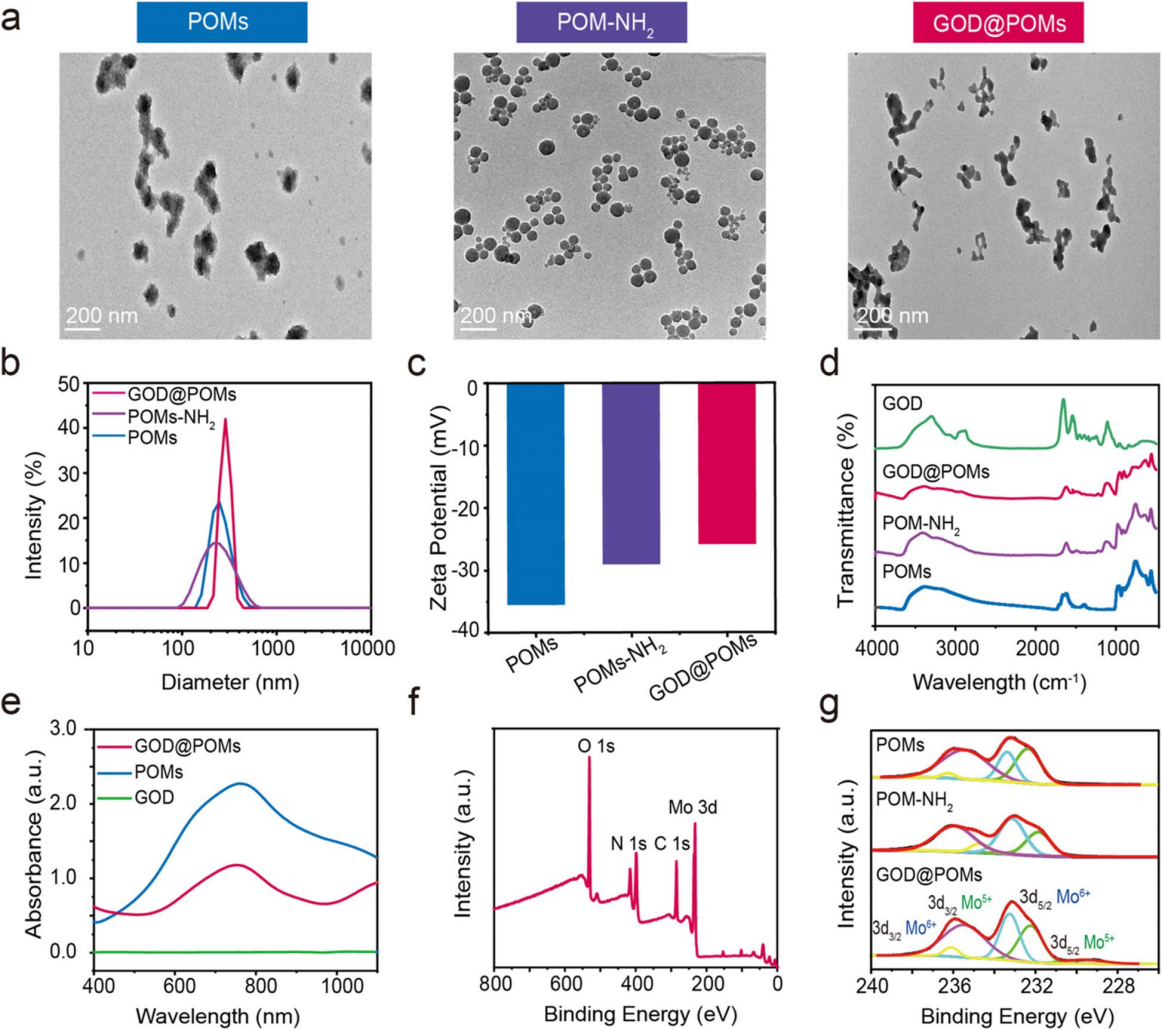
**a** TEM images of POMs, POM-NH_2_ and GOD@POMs. **b** Size distribution of POMs, POM-NH_2_ and GOD@POMs in water. **c** Zeta potential of POMs, POM-NH_2_ and GOD@POMs in water. **d** FTIR spectra of GOD, POMs, POM-NH_2_ and GOD@POMs. **e** UV-vis-NIR spectra of POMs, GOD and GOD@POMs. **f** Wide scan XPS spectra of GOD@POMs. **g** XPS spectra of Mo 3d in POMs, POM-NH_2_ and GOD@POMs

For the successful synthesis of Mo-based POMs, Mo_2_C powder was reacted with H_2_O_2_ in a facile one-pot procedure. As shown in the TEM images, the obtained POMs possess a spherical structure (Fig. 1a). Subsequently, for the GOD grafting, the POMs were reacted with APTES to generate amino groups onto the surface, termed as POM-NH_2_ (Fig. 1a), the average diameter of which is 215 nm, and then covalently conjugated with the carboxyl of GOD through amide bond via EDC/NHS coupling, defined as GOD@POMs. Next, TEM images show that GOD@POMs feature spherical shape and the average diameter is around 255 nm (Fig. 1a, b). Besides, the zeta potential slightly increases from POMs (− 35.5 mV) to POM-NH_2_ (− 29.0 mV) to GOD@POMs (− 25.8 mV), which originates from the surface GOD modification (Fig. 1c). FTIR spectroscopy analysis indicates the vibration bands at 3300–3500 cm^− 1^ region related to the stretching vibration of -NH_2_ and the band centered at 1650 cm^− 1^ ascribed to the stretching vibration of C=O (Fig. 1d), confirming the successful surface conjugation of GOD. Notably, the loading amount of GOD is tested and calculated to be 10.0 wt% according to the thermogravimetric analysis (TGA) results (Fig. S1, Supporting Information). The UV-vis-NIR absorption spectrum of GOD@POMs exhibits broad and intense absorption in NIR region, especially in NIR-II biowindow, which indicates their high potential to serve as the desirable photothermal agent (Fig. 1e). In addition, XPS spectra of the elements O, N, C, and Mo further demonstrate the desirable synthesis of GOD@POMs (Fig. 1f). Especially, the Mo element in POMs displays mixed valence states containing Mo^5+^ and Mo^6+^ on the 3d orbit (Fig. 1g). Following amino group and GOD surface modification, the mixed valence states of Mo in POM-NH_2_ and GOD@POMs reveal neglectable change. Through the observation of UV-vis-NIR, the absorbance of GOD@POMs exhibits neglectable changes with the prolongation of time, no matter it was dispersed in water or in PBS, indicating that it features preferable solution stability (Fig. S2, Supporting Information).

### Catalytic and photothermal performance of GOD@POMs

**Fig. 2:**
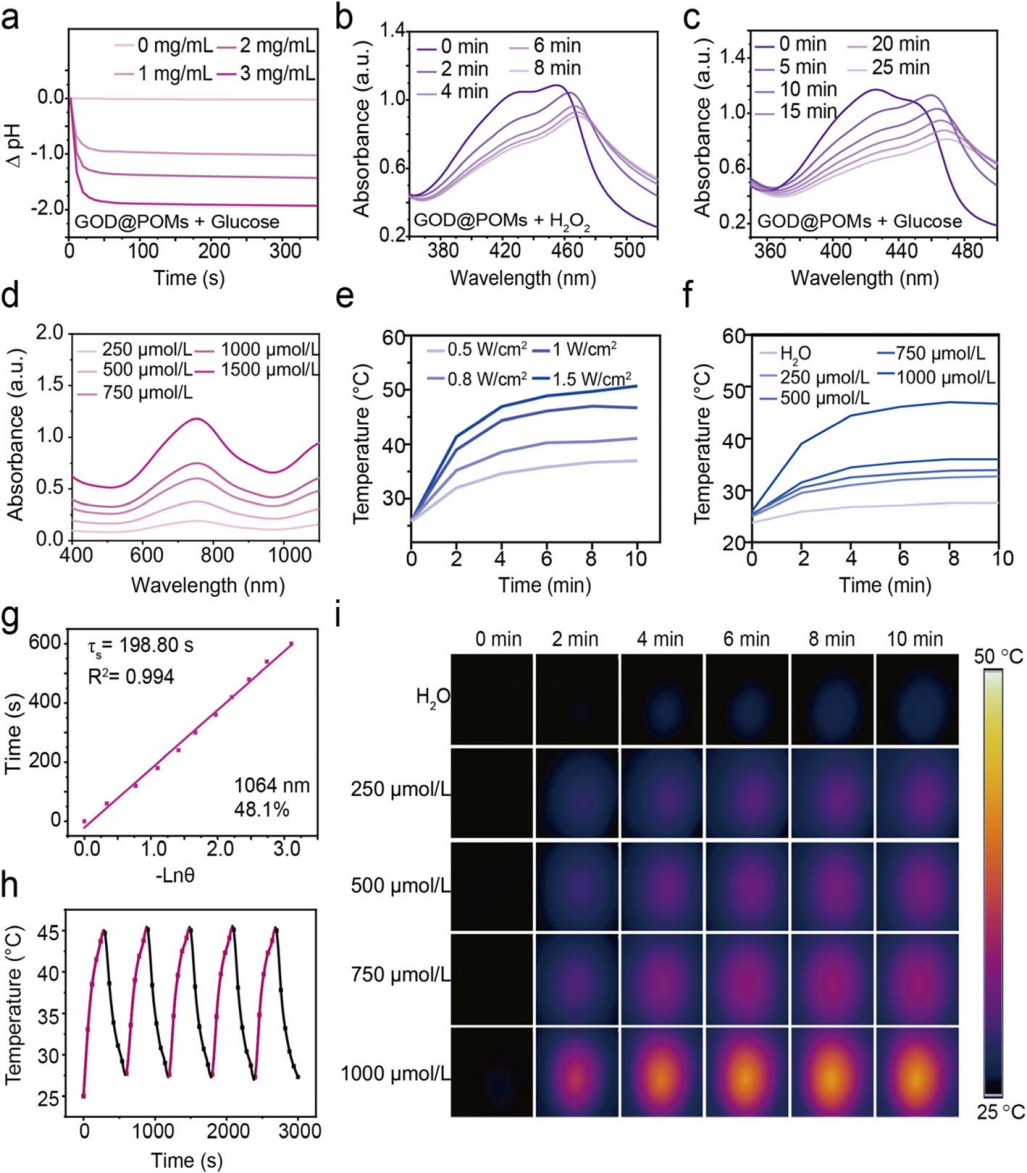
**a** pH value changes after GOD@POMs treatment with glucose at various concentrations. **b** Time-dependent UV-vis-NIR spectra of the mixture containing DPBF, GOD@POMs and H_2_O_2_ (10 mM). **c** Time-dependent UV-vis-NIR spectra of the mixture containing DPBF, GOD@POMs and glucose (1 mg/mL). **d** UV-vis-NIR spectra of GOD@POMs at various concentrations. **e** Photothermal heating curves of GOD@POMs dispersion under 1064 nm laser irradiation with different power densities as a function of time. **f** Photothermal heating curves of GOD@POMs dispersion at different concentrations under 1064 nm laser irradiation (1 W/cm^2^) as a function of time. **g** Calculation of the photothermal-conversion efficiency of GOD@POMs dispersion under 1064 nm laser irradiation. Time constant (τ_s_) for heat transfer from the system by applying the linear time data from the cooling period. **h** Photothermal stability measurements of GOD@POMs dispersion under five on/off 1064 nm laser cycles irradiation (1 W/cm^2^). **i** Infrared thermal images of GOD@POMs aqueous dispersion with diverse concentrations under 1064 nm irradiation (1 W/cm^2^) at designed time points

Given that GOD can efficiently catalyze glucose into gluconic acid and H_2_O_2_, the GOD@POMs were reacted with glucose to testify the catalytic activity of conjugated GOD. The results indicate that the generated gluconic acid induces a significant decrease in pH value, which is in a glucose concentration-dependent manner (Fig. 2a). Notably, after treatment with glucose and the decrease of pH induced by generated gluconic acid, the TEM images graphically reveal the aggregation of POMs in acidic environments, showing TME-specific accumulation (Fig. S3, Supporting Information). Besides, under the same concentration, the blue color of GOD@POMs dispersion deepens monotonically in acidic environment, corresponding to the significantly elevated UV-vis-NIR absorption (Fig. S4, Supporting Information). Subsequently, DPBF was applied to detect ^1^O_2_ generation capability of GOD@POMs. As the H_2_O_2_ co-reaction time prolonged, the characteristic absorption peak of DPBF at 420 nm significantly decreased (Fig. 2b), indicating the substantial ^1^O_2_ production. Furthermore, once the addition of glucose, the absorption peak of DPBF decreases in both time-dependent and glucose concentration-dependent manners (Fig. 2c, Fig. S5, Supporting Information), which is attributed to the presence of GOD in GOD@POMs catalyzing the oxidation of glucose into H_2_O_2_, thus initiating a cascade catalytic reaction for ^1^O_2_ generation.

In addition, the UV-vis-NIR absorption spectra reveals that GOD@POMs feature wide and strong absorption covering both the NIR-I and NIR-II biological window (Fig. 2d), elucidating the strong NIR laser absorption ability of GOD@POMs, following a concentration-dependent manner. Then, the photothermal performance of GOD@POMs was assessed. As expected, after exposure to 1064 nm laser irradiation, the temperature of GOD@POMs aqueous solution remarkably rises in a power density-dependent and concentration-dependent way (Fig. 2e, f and i), and the temperature can reach 50.7 °C when Mo concentration is 1000 μmol/L under 1064 nm laser irradiation at 1.5 W/cm^2^ for 10 min. The photothermal-conversion efficiency of GOD@POMs is calculated to be 48.1% at 1064 nm (Fig. 2g, Fig. S6Supporting Information), surpassing most available photothermal agents such as MnSe_2_@PVP (39.1%) (Table S1). In addition, almost no apparent deterioration can be detected during the five successive laser on/off cycles, confirming the impressive photothermal stability of GOD@POMs (Fig. 2h).

### In vitro photothermal-enhanced cascade catalytic therapy

**Fig. 3:**
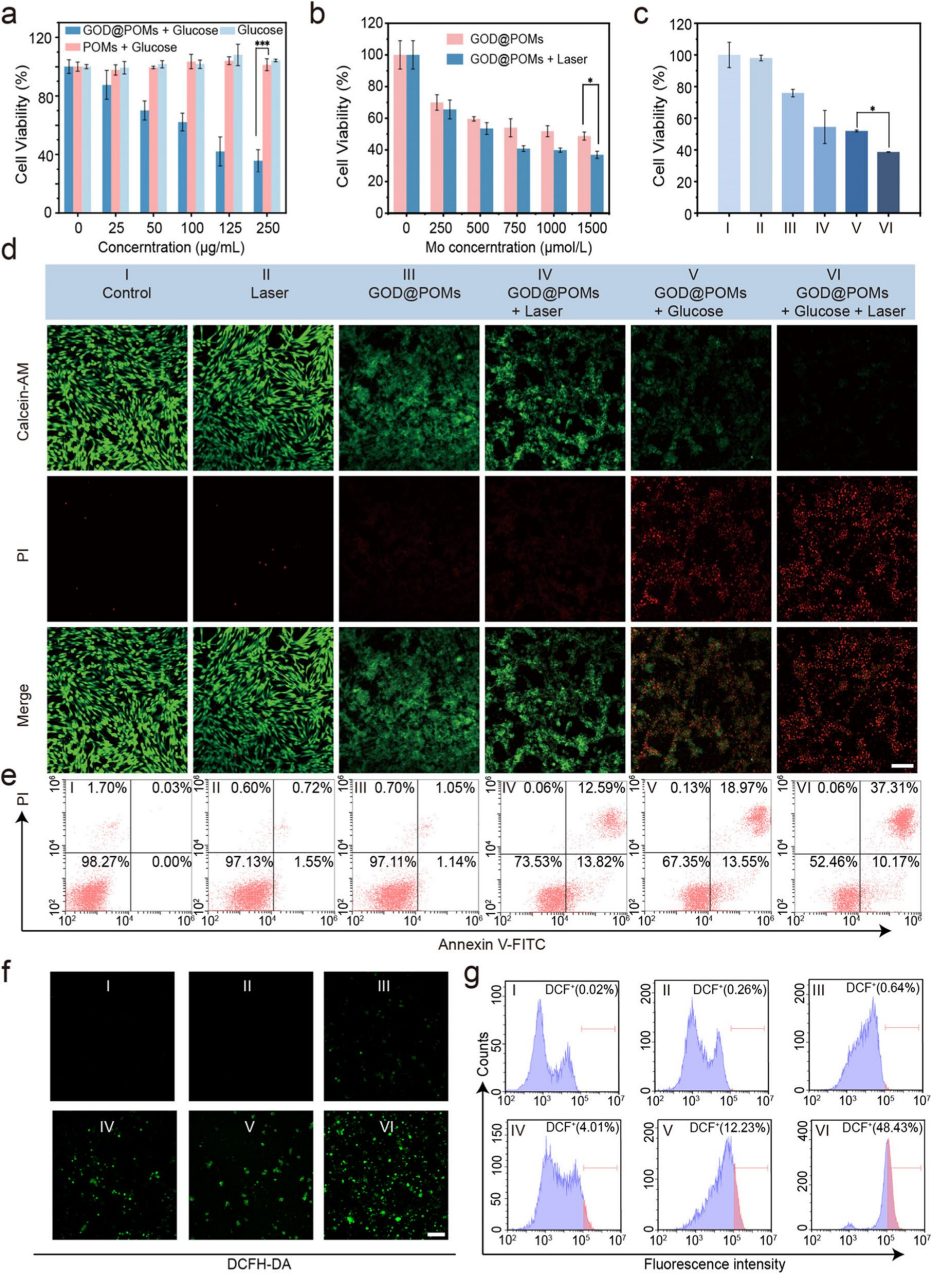
**a** Cell viabilities of C6 cells after treatment with various concentrations of glucose with or without GOD@POMs or POMs. **b** Cell viabilities of C6 cells after treatment with GOD@POMs at various concentrations with or without laser irradiation (1064 nm, 1 W/cm^2^, 5 min). **c** Cell viabilities of C6 cells after different treatments: (I) Control; (II) Laser; (III) GOD@POMs; (IV) GOD@POMs + Laser; (V) GOD@POMs + Glucose; (VI) GOD@POMs + Glucose + Laser. **d** CLSM images of C6 cells stained with Calcein AM and PI after varied treatments. **e** Flow cytometry apoptosis assay of C6 cells stained by Annexin-FITC and PI after varied treatments. **f** CLSM images and flow cytometry of intracellular ROS production of C6 cells stained with DCFH-DA after different treatments. Scale bar: 100 μm. ***p < 0.001, **p < 0.01 and *p < 0.05

The cellular uptake of C6 cells towards GOD@POMs was performed to uncover their internalization behaviour. To verify the cellular uptake of GOD@POMs, the FITC-labelled GOD@POMs were incubated with C6 cells. After 8 h co-incubation, the stronger green fluorescence intensity can be clearly observed, which indicates that the GOD@POMs can be effectively internalized by the endocytic pathway (Fig. S7, Supporting Information). Prior to investigate the anti-cancer effects of GOD@POMs in vitro, the cytotoxicity of GOD@POMs was initially assessed on L929 murine fibroblast cell line using standard CCK-8 assay. After co-incubation at various Mo concentrations for 24 h, GOD@POMs exhibit no obvious cytotoxicity to L929 cells, and the cell viability is still above 80% even exposure to nanoparticles at the Mo concentration of as high as 2000 μmol/L (Fig. S8, Supporting Information), indicating that GOD@POMs possess low toxicity to normal cells. Subsequently, the in vitro therapeutic efficacy of GOD@POMs was evaluated on C6 rat glioma cell line. As shown in Fig. 3a, compared to glucose only group and POMs + Glucose group which exhibit no obvious cell-killing abilities, with the glucose concentration increasing from 0 to 250 μg/mL, the cell viability of GOD@POMs + Glucose group declines significantly from 100 to 35.7%, revealing that GOD@POMs react with glucose to produce ROS for cell killing through cascade catalytic reaction. Moreover, under 1064 nm laser irradiation, in contrast to only 43.4% of apoptotic cells in the POMs group, GOD@POMs induce up to 63.1% C6 cell death at the Mo concentration of 1500 μmol/L, which is lower than the group without laser irradiation and POMs + Laser group. The superior cell killing of GOD@POMs under laser irradiation is owing to the acidity environment enhanced by generated gluconic acid, making the POMs aggregate and resulting stronger hyperthermia effects (Fig. 3b, Fig. S9, Supporting Information).

To further confirm the synergistic therapy effect, C6 cells were divided into six groups: (I) Control; (II) Laser (1 W/cm^2^, 5 min); (III) GOD@POMs; (IV) GOD@POMs + Laser (1 W/cm^2^, 5 min); (V) GOD@POMs + Glucose; (VI) GOD@POMs + Glucose + Laser (1 W/cm^2^, 5 min). Comparatively, there are 52.0% and 54.5% cells survived in the GOD@POMs + Laser group and GOD@POMs + Glucose group, respectively, while only 38.69% of C6 cells survived in the GOD@POMs + Glucose + Laser group, verifying the synergistic anti-cancer effect in vitro (Fig. 3c). Furthermore, the killing effect after different treatments is detected by co-staining with calcein AM (green, live cells) and propidium iodide (PI, red, dead cells) (Fig. 3d). The results show that, compared with the groups without glucose, GOD@POMs + Glucose group and GOD@POMs + Glucose + Laser group display strong red fluorescence from PI, resulting from the GOD-induced starvation therapy by glucose consumption following the H_2_O_2_ generation-facilitated Mo-based CDT. Moreover, there is almost no green fluorescence from calcein AM in GOD@POMs + Glucose + Laser group, which is ascribed to the PTT and meanwhile the photonic hyperthermia-promoted cascade catalytic reaction, achieving synergistic therapy. Corresponding to the results of CLSM images, the flow cytometry apoptosis assay exhibits that the GOD@POMs + Glucose + Laser group causes the maximum amounts of total 47.48% cell apoptosis (Fig. 3e). Moreover, in order to explore the intracellular mechanism mainly depending on the ROS, DCFH-DA as a typical ROS fluorescent probe was used (Fig. 3f). Compared with GOD@POMs group, C6 cells in GOD@POMs + Glucose group exhibit enhanced green fluorescence by CLSM images and flow cytometry analysis, which indicates that GOD@POMs react with glucose to yield ROS. Besides, the cells after treatment with GOD@POM + Glucose + Laser display the strongest green fluorescence, suggesting that the hyperthermia can accelerate the ROS generation (Fig. 3g).

### In vivo anti-tumor effect of GOD@POMs on the C6 tumor-bearing mice

Considering that the biosafety is the first priority for the biomedical application, we systematically studied the biocompatibility and biodistribution of GOD@POMs in vivo. The Kunming mice were intravenously injected with GOD@POMs and the blood and main organs were collected including heart, liver, spleen, lung and kidney for biosafety assessment. During the 4 weeks observation, compared with control group, no significant difference in body weight is monitored in the mice after treatment with GOD@POMs (Fig. S10, Supporting Information). Besides, after administration with GOD@POMs, the blood routine is distributed within the normal range, suggesting that GOD@POMs induce no side effects to the mice (Fig. S11, Supporting Information). Additionally, almost no obvious pathological abnormality is observed in the GOD@POMs group (Fig. S12, Supporting Information). All these in vivo evaluation data strongly demonstrate the desirable biosafety of the engineered multifunctional GOD@POMs nanosystem.

To evaluate the in vivo behaviors, Cy5.5 labelled GOD@POMs were fabricated for biodistribution and tumor accumulation study using fluorescent imaging. As shown in Fig. S13a and S13b, the fluorescence intensity of the tumor gradually increases and reaches the peak after 4 h, indicating the efficient tumor uptake of GOD@POMs. At the following time points, the fluorescence signals slowly decrease. Moreover, the ex vivo fluorescence images and corresponding fluorescence intensity illustrate that the GOD@POMs could efficiently accumulate in the tumor and clear from other organs. (Fig. S13c-d, Supporting Information).

**Fig. 4:**
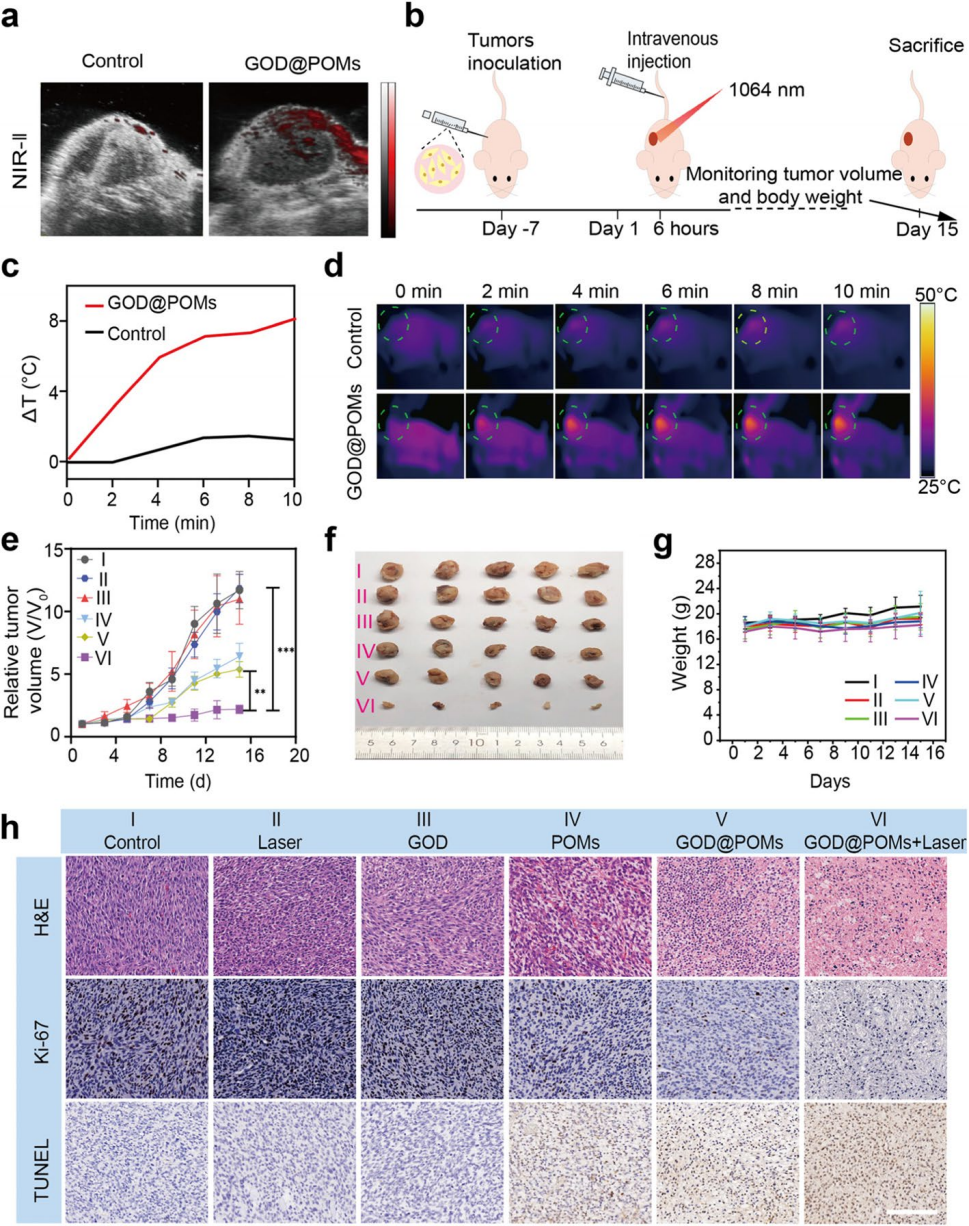
**a** In vivo PA images of C6 tumor-bearing mice before and after intravenous injection of GOD@POMs. **b** Schematic illustration of the C6 tumor-bearing mice model establishment and the therapeutic process. **c** Photothermal heating curves and (**d**) corresponding IR thermal images of C6 tumor-bearing mice intravenously injected with PBS or GOD@POMs under laser irradiation (1064 nm, 1 W/cm^2^). **e** Relative tumor volume growth curves on C6 tumor-bearing mice after different treatments, including (I) Control, (II) Laser, (III) GOD, (IV) POMs, (V) GOD@POMs and (VI) GOD@POMs + Laser. **f** Digital photos of resected tumors from C6 tumor-bearing mice after different treatments. **g** Body weights of C6 tumor-bearing mice after different treatments. **h** H&E, Ki67 and TUNEL analysis of tumor tissues collected after various treatments. Scale bar: 100 μm. ***p < 0.001, **p < 0.01 and *p < 0.05

Inspired by the excellent therapeutic effects in vitro, the satisfactory biosafety in vivo and efficient tumor accumulation, we further investigated the anti-tumor efficacy of GOD@POMs on C6 tumor-bearing BALB/C nude mice. Since GOD@POMs possess the strong absorption in both NIR-I and NIR-II regions, they can serve as a potential contrast agent for PA imaging in vivo. Expectably, with the increased Mo concentration, the PA signals are gradually enhanced in NIR biological window, especially in NIR-II (Fig. S14, Supporting Information). After intravenous injection of GOD@POMs, apparent PA signals are observed in the tumor site, which illustrates that GOD@POMs not only can act as a remarkable contrast-enhanced NIR-II PA agent but also can effectively gather at tumor site through the typical enhanced permeability and retention (EPR) effect (Fig. 4a). Subsequently, all C6-tumor-bearing mice were randomly divided into 6 groups: (I) Control, (II) Laser, (III) GOD, (IV) POMs, (V) GOD@POMs and (VI) GOD@POMs + Laser, in which the mice in group (II) and (VI) are irradiated with 1064 nm laser (1 W/cm^2^) for 10 min after GOD@POMs injection (Fig. 4b). As shown in Fig. 4c, the temperature of tumor in GOD@POMs + Laser group quickly increases 8 °C within 10 minutes, whereas the temperature in laser alone group no significant increase (Fig. 4d), which manifests the benign photothermal performance of GOD@POMs. During the treatment process, the mice weight and tumor volume are recorded every 2 days. Compared with the control group, GOD@POMs group exhibits a certain tumor growth inhibition ratio of 50% owing to the GOD-mediated starvation therapy combined with POM-based CDT. Important, it is found that the tumor growth decreases to only 20% in GOD@POMs + Laser group, confirming that PTT can further improve therapeutic effects (Fig. 4e, f). The mice in each group shows negligible change in body weight, demonstrating almost no adverse effects during the treatment (Fig. 4g).

Subsequently, the tumor sections of each group are stained with the H&E histological analysis, Ki67 and TUNEL antibody staining for pathological evaluation. In accordance with relative tumor volume curves, GOD@POMs + Laser group shows the most significant cell necrosis or damage, in which large areas of tumor cells appear nuclear solid fragmentation and pale red cytoplasm in H&E staining. The results are further confirmed by a large number of apoptotic tumor cells in GOD@POMs + Laser group stained by TUNEL and the obviously reduced proliferation of tumor cells stained by Ki-67 (Fig. 4h). Additionally, in each group, there is no obvious damage or inflammation in H&E staining image of major tissues (e.g., heart, liver, spleen, lung and kidney) (Fig. S15, Supporting Information), suggesting the high therapeutic biosafety of GOD@POMs during the treatment.

### RNA sequencing and analysis of C6 tumor-bearing mice administrated with GOD@POMs under NIR-II laser irradiation

**Fig. 5:**
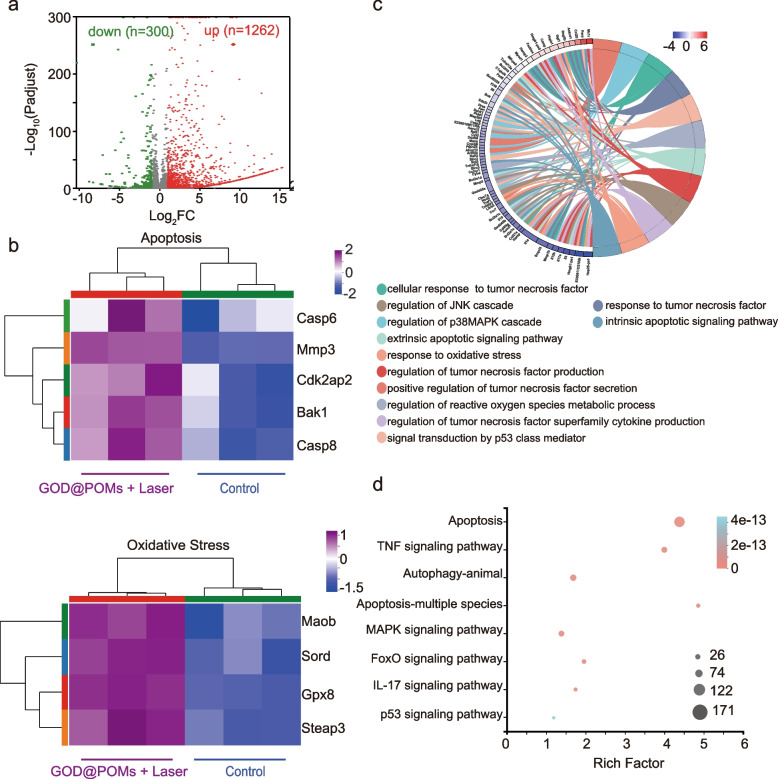
**a** Volcano plot of differentially regulated genes between control group and GOD@POMs + laser group. **b** Heat-map of differentially expressed genes associated with apoptosis and oxidative stress. **c** GO enrichment analysis of the different genes after the GOD@POMs + laser treatment. **d** KEGG pathway enrichment analysis based on RNAseq after the GOD@POMs + laser treatment.

To further investigate the underlying mechanism of GOD@POMs-mediated synergistic antitumor effect, the typical RNA sequencing (RNAseq) was carried out. As shown in volcano plot, compared with control group, there are 1562 differentially expressed genes, including 1262 up-regulated genes and 300 down-regulated genes (Fig. 5a). Among them, apoptosis-related genes, such as Casp6, Bak1 and Casp8 are up-regulated, suggesting that the GOD@POMs + laser treatment could cause cancer cells apoptosis (Fig. 5b). Meanwhile, the up-regulated expression of genes, including Maob, Sord, Steap3 and Gpx8 are associated with oxidative stress, illustrating that the produced ROS plays an important role in tumor growth inhibition (Fig. 5b). Subsequently, in order to understand the biological processes and molecular metabolic pathways, Gene Ontology (GO) analysis and Kyoto Encyclopedia of Genes and Genomes (KEGG) pathway analysis were performed to analyze the genes set enrichment. GO analysis indicates that up-regulated genes in GOD@POMs + laser group are related to the regulation of tumor necrosis (Fig. 5c). In addition, after GOD@POMs + laser treatment, KEGG analysis shows that differentially expressed genes are concentrated in pathways associated with apoptosis, tumor necrosis factor (TNF) and mitogen-activated protein kinase (MAPK) signaling pathway (Fig. 5d). Therefore, the RNA sequencing data further demonstrates that the GOD@POMs + laser treatment produces anti-tumor effects associating with tumor apoptosis and necrosis.

## Discussion

Compared with normal cells, cancer cells prefer to utilize aerobic glycolysis rather than oxidative phosphorylation for adenosine triphosphate (ATP) production, inducing an increased glucose import to satisfy their rapid proliferation and energy demand. Accordingly, starvation therapy is developed as an effective strategy to suppress tumor growth by blocking glucose supply. GOD, as an endogenous oxido-reductase, can efficiently catalyze glucose into H_2_O_2_ and gluconic acid in the presence of molecular oxygen thereby depriving glucose and increasing acidity as well as H_2_O_2_ content in TME. The overproduced H_2_O_2_ not only trigger oxidative stress but also can be applied as the substrate of Fenton/Fenton-like reaction for ROS generation. Unlike traditional Fenton/Fenton-like agents, based on the specific electronic structure, Mo-based POM is sensitive to the stimulus of pH and redox environment such as GSH. Mo (VI) to Mo (V) reduction with the help of GSH renders the ^1^O_2_ production catalyzing H_2_O_2_ decomposition through Russell mechanism [36].

In this study, we engineered a tumor-specific catalytic nanosystem GOD@POMs realizing PTT-augmented synergistic starvation combined with CDT. On the basis of POM-mediated CDT and acid-induced aggregation for PTT, the GOD-mediated glucose consumption can realize H_2_O_2_ self-supply and more acidic TME, which further promote CDT and PTT process. Moreover, the increased temperature caused by PTT can elevate the reaction activity of GOD, thus building a paradigm for interlocking synergistic anti-tumor pattern.

In spite of the therapeutic effect of GOD@POMs is only preliminarily evaluated on C6 cancer cell and C6 derived cell line xenograft (CDX) model, our work has suggested that GOD@POMs can substantially inhibit tumor growth. In the future, it will be interesting to test whether it can be extended to other tumor models or patient derived xenograft (PDX) to comprehensively prove its anti-tumor effect and clinical transformation potential. Additionally, only Mo-based POM is used to covalently connect GOD in current experiment, and it is also worth further exploring whether GOD or other endogenous oxido-reductase could be combined with other pre-transition metal-based POMs such as vanadium, tungsten and so on to produce similar effects, which will broaden the application of POM in biomedical industry.

## Conclusions

In summary, we have successfully designed and engineered a highly synergistic therapeutic nanoplatform GOD@POMs by elaborately grafting GOD onto POM. GOD catalyzes the oxidation of intratumor glucose into gluconic acid and H_2_O_2_, which not only blocks the tumor energy supply for starvation therapy, but also elevates H_2_O_2_ level for subsequent CDT. Furthermore, the redox reaction between Mo (VI) sites and Mo(V) active sites on POM leads to GSH depletion and then trigger the self-supply H_2_O_2_ to yield abundant ^1^O_2_ by Mo-mediated Russell reaction. Meanwhile, the decreased pH in TME resulting from the produced gluconic acid promotes the aggregation of Mo-based POM, which can act as NIR-II PTAs for PA imaging-guided photonic tumor hyperthermia, further augmenting the nanocatalytic tumor-therapeutic efficacy. This specific tumor-specific nanocatalytic paradigm represents the desirable tumor treatment modality with concurrently high therapeutic efficacy and biosafety.

## Supplementary Information

**Additional file 1: Fig. S1.** TG analysis of POMs, POM-NH_2_ and GOD@POMs. **Fig. S2.** UV-vis-NIR absorption of GOD@POMs dispersed in H_2_O or PBS at 24  and 72 h. **Fig. S3.** TEM image of aggregated GOD@POMs after reaction with glucose. **Fig. S4.** UV-vis-NIR spectra and the corresponding digital photo of GOD@POMs dispersed in PBS with different pH values ranging from 4.0 to 7.3. **Fig. S5.** Glucose concentration and time-dependent oxidation of DPBF by GOD@POMs. **Fig. S6.** Heating and cooling curve of GOD@POMs aqueous solution under 1064 nm laser irradiation at 1.0 W/cm^2^. **Fig. S7.** CLSM images of C6 cells incubated with FITC-labelled GOD@POM after 8 h. **Fig. S8.** Cell viability assay of L929 cells after treatment with GOD@POMs 24 h at various concentrations. **Fig. S9.** Cell viabilities of C6 cells after treatment with POMs at various concentrations with or without laser irradiation (1064 nm, 1 W/cm^2^, 5 min). **Fig. S10.** The body weight of Kunming mice during 28 days observation after different treatments. **Fig. S11.** Biosafety evaluations of GOD@POMs in vivo*.* **Fig. S12.** H&E staining of major organ including heart, liver, spleen, lung and kidney collected from Kunming mice after 28 days treatment. Scale bar: 100 μm. **Fig. S13.** (a) Representative in vivo fluorescence images of C6 tumor-bearing mice at 0, 2, 4, 6, 8 and 12 h after intravenous injection of GOD@POMs. (b) Quantitative ROI assays of the fluorescence intensity of tumor at designated time points. (c) Ex vivo fluorescence images of major organs at 12 and 24 h after intravenous injection of GOD@POMs (H: heart, Li: liver, Sp: spleen, Lu: lung, Ki: kidney, Tu: tumor). (d) Quantitative ROI assays of the ex vivo fluorescence intensity of major organs and tumors. **Fig. S14.** In vitro NIR-I and NIR-II PA images of GOD@POMs at various Mo concentration (250, 500, 750, 1000 and 1500 μmol/L). **Fig. S15.** H&E staining of major organ tissues collected from mice after different treatments Scale bar: 100 μm. **Table S1.** The light-to-heat conversion efficiency of different of photothermal agents in the reported literatures.

## Data Availability

The data and analysis generated in this study are included in articles and supplementary information files.

## Abbreviations

*CDT*: Chemodynamic therapy
*POM*: Polyoxometalate
*H*_*2*_*O*_*2*_: Hydrogen peroxide
*ROS*: Reactive oxygen species
*TME*: Tumor microenvironment
*GSH*: Glutathione
*GOD*: Glucose oxidase
*PTT*: Photothermal therapy
*PTAs*: Photothermal agents
*NIR*: Near-infrared
*PA*: Photoacoustic
^*1*^*O*_*2*_: Singlet oxygen
*APTES*: 3-aminopropyltriethoxysilane
*EDC*: 1-(3-Dim-ethylaminopropyl)-3-ethylcarbodiimide hydrochloride
*NHS*: N-Hydroxy succinimide
*DPBF*: 1, 3-diphenylisobenzofuran
*DCFH-DA*: 2,7-dichlorofluorescein diacetate
*FBS*: Fetal bovine serum
*PBS*: Phosphate buffer saline
*SEM*: Scanning electron microscope
*TEM*: Transmission electron microscope
*UV–vis–NIR*: Ultraviolet-visible-near-infrared
*FTIR*: Fourier transform infrared
*XPS*: X-ray photoelectron spectroscopy
*TGA*: Thermogravimetric analysis
*EPR*: Enhanced permeability and retention effect
*CCK-8*: Cell counting kit-8
*H&E*: hematoxylin and eosin
