# Supplementary material for: Engineering tumor-specific catalytic nanosystem for NIR-II photothermal-augmented and synergistic starvation/chemodynamic nanotherapy
Source: Biomater Res. 2022 Nov 26;26:66. doi: 10.1186/s40824-022-00317-y (PMC9701438; doi:10.1186/s40824-022-00317-y)
Supplement: Supplementary file 1 — Additional file 1: Fig. S1. TG analysis of POMs, POM-NH2 and GOD@POMs. Fig. S2. UV-vis-NIR absorption of GOD@POMs dispersed in H2O or PBS at 24 and 72 h. Fig. S3. TEM image of aggregated GOD@POMs after reaction with glucose. Fig. S4. UV-vis-NIR spectra and the corresponding digital photo of GOD@POMs dispersed in PBS with different pH values ranging from 4.0 to 7.3. Fig. S5. Glucose concentration and time-dependent oxidation of DPBF by GOD@POMs. Fig. S6. Heating and cooling curve of GOD@POMs aqueous solution under 1064 nm laser irradiation at 1.0 W/cm2. Fig. S7. CLSM images of C6 cells incubated with FITC-labelled GOD@POM after 8 h. Fig. S8. Cell viability assay of L929 cells after treatment with GOD@POMs 24 h at various concentrations. Fig. S9. Cell viabilities of C6 cells after treatment with POMs at various concentrations with or without laser irradiation (1064 nm, 1 W/cm2, 5 min). Fig. S10. The body weight of Kunming mice during 28 days observation after different treatments. Fig. S11. Biosafety evaluations of GOD@POMs in vivo. Fig. S12. H&E staining of major organ including heart, liver, spleen, lung and kidney collected from Kunming mice after 28 days treatment. Scale bar: 100 μm. Fig. S13. (a) Representative in vivo fluorescence images of C6 tumor-bearing mice at 0, 2, 4, 6, 8 and 12 h after intravenous injection of GOD@POMs. (b) Quantitative ROI assays of the fluorescence intensity of tumor at designated time points. (c) Ex vivo fluorescence images of major organs at 12 and 24 h after intravenous injection of GOD@POMs (H: heart, Li: liver, Sp: spleen, Lu: lung, Ki: kidney, Tu: tumor). (d) Quantitative ROI assays of the ex vivo fluorescence intensity of major organs and tumors. Fig. S14. In vitro NIR-I and NIR-II PA images of GOD@POMs at various Mo concentration (250, 500, 750, 1000 and 1500 μmol/L). Fig. S15. H&E staining of major organ tissues collected from mice after different treatments Scale bar: 100 μm. Table S1. The light-to-heat conversion efficiency [file 40824_2022_317_MOESM1_ESM.docx]

**Supporting information**

**Engineering tumor-specific catalytic nanosystem for NIR-II photothermal-augmented and synergistic starvation/chemodynamic nanotherapy**

Shuixiu Zhou^a, 1^, Jiahuan Xu^a, 1^, Yanfei Dai^c, 1^, Yan Wei^a^, Liang Chen^b^, Wei Feng^b, *^, Yu Chen^b, *^ and Xuejun Ni^a, *^

^a^ Department of Medical Ultrasound, Affiliated Hospital of Nantong University, Nantong 226001, P. R. China. Email: dyfnxj213@163.com.

^b^ Materdicine Lab, School of Life Sciences, Shanghai University, Shanghai 200444, P. R. China Email: fengw@shu.edu.cn; chenyuedu@shu.edu.cn.

^c^ Radiology Department, Branch of Affiliated Hospital of Nantong University, Nantong 226001, P. R. China.


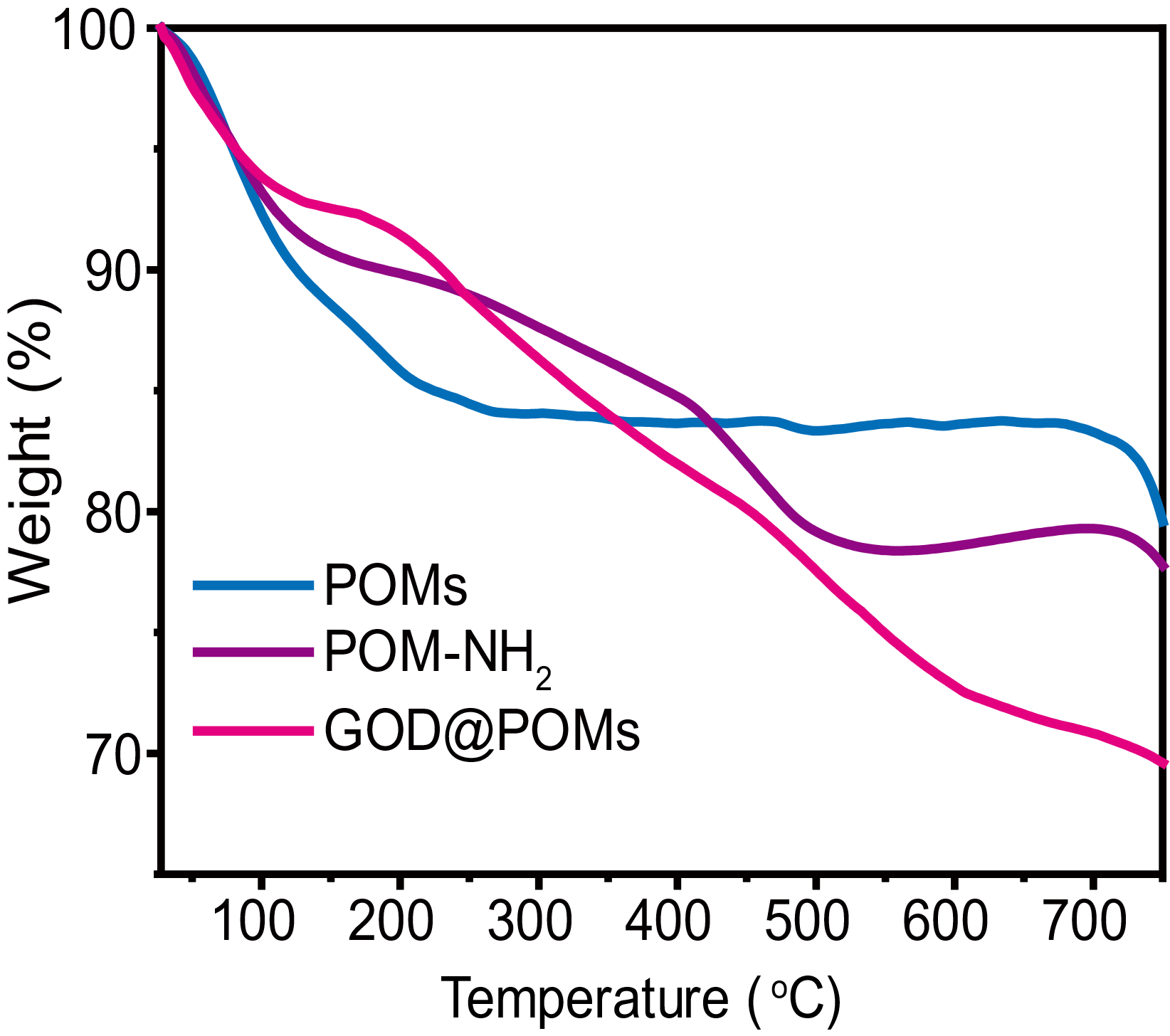


**Figure S1.** TG analysis of POMs, POM-NH_2_ and GOD@POMs.

**
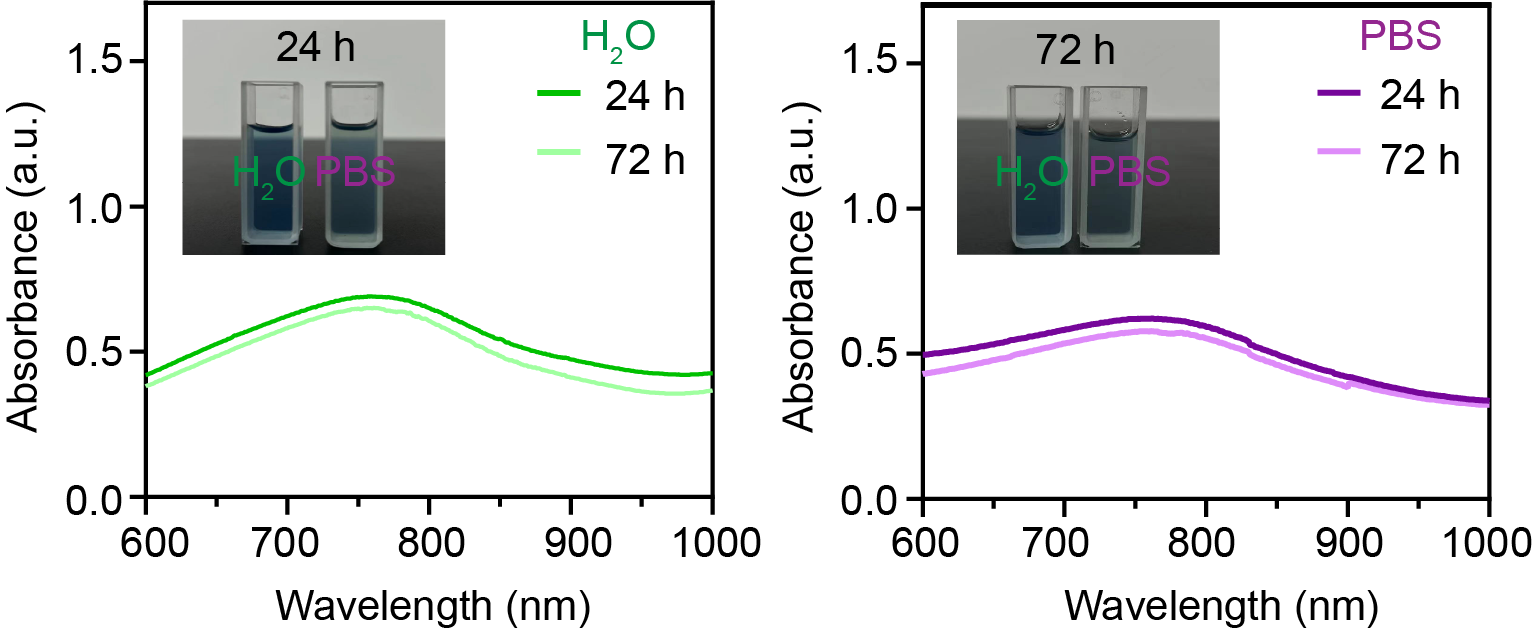
**

**Figure S2.** UV-vis-NIR absorption of GOD@POMs dispersed in H_2_O or PBS at 24 and 72 h.


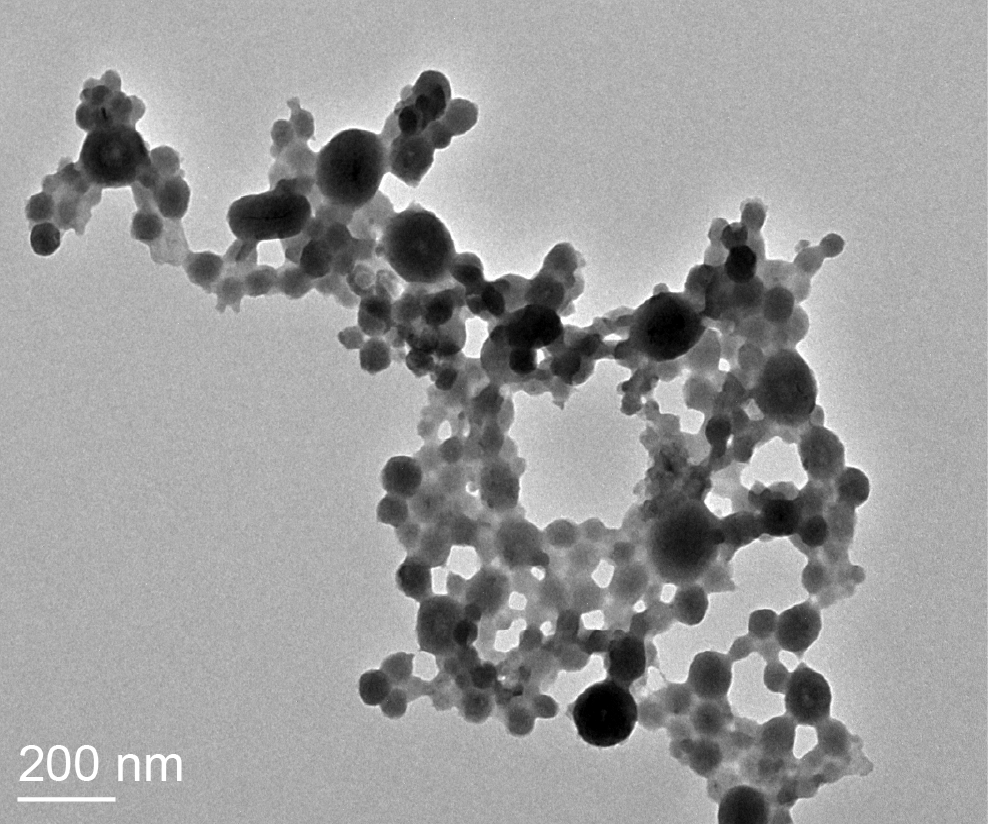


**Figure S3.** TEM image of aggregated GOD@POMs after reaction with glucose.


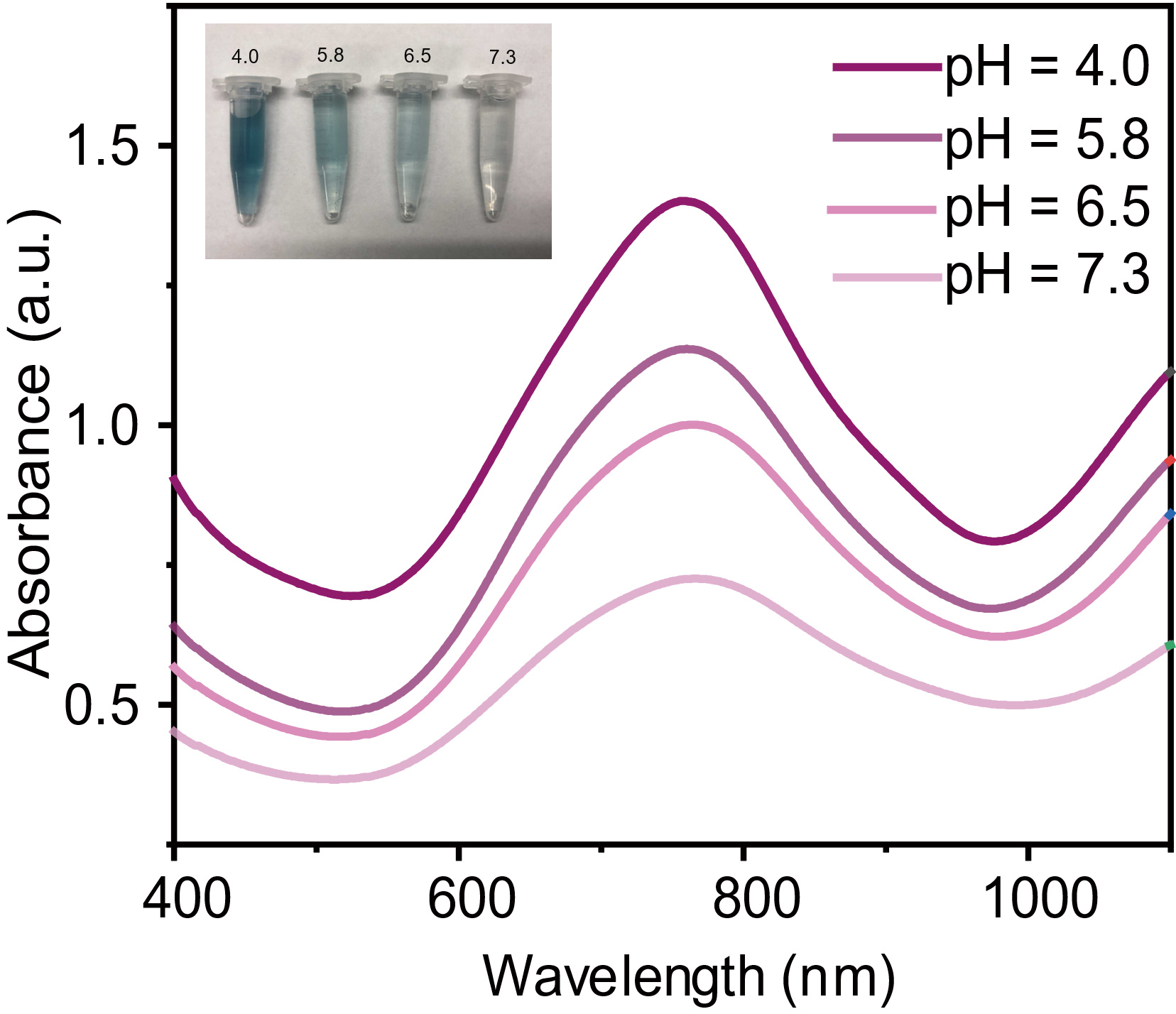


**Figure S4.** UV-vis-NIR spectra and the corresponding digital photo of GOD@POMs dispersed in PBS with different pH values ranging from 4.0 to 7.3.


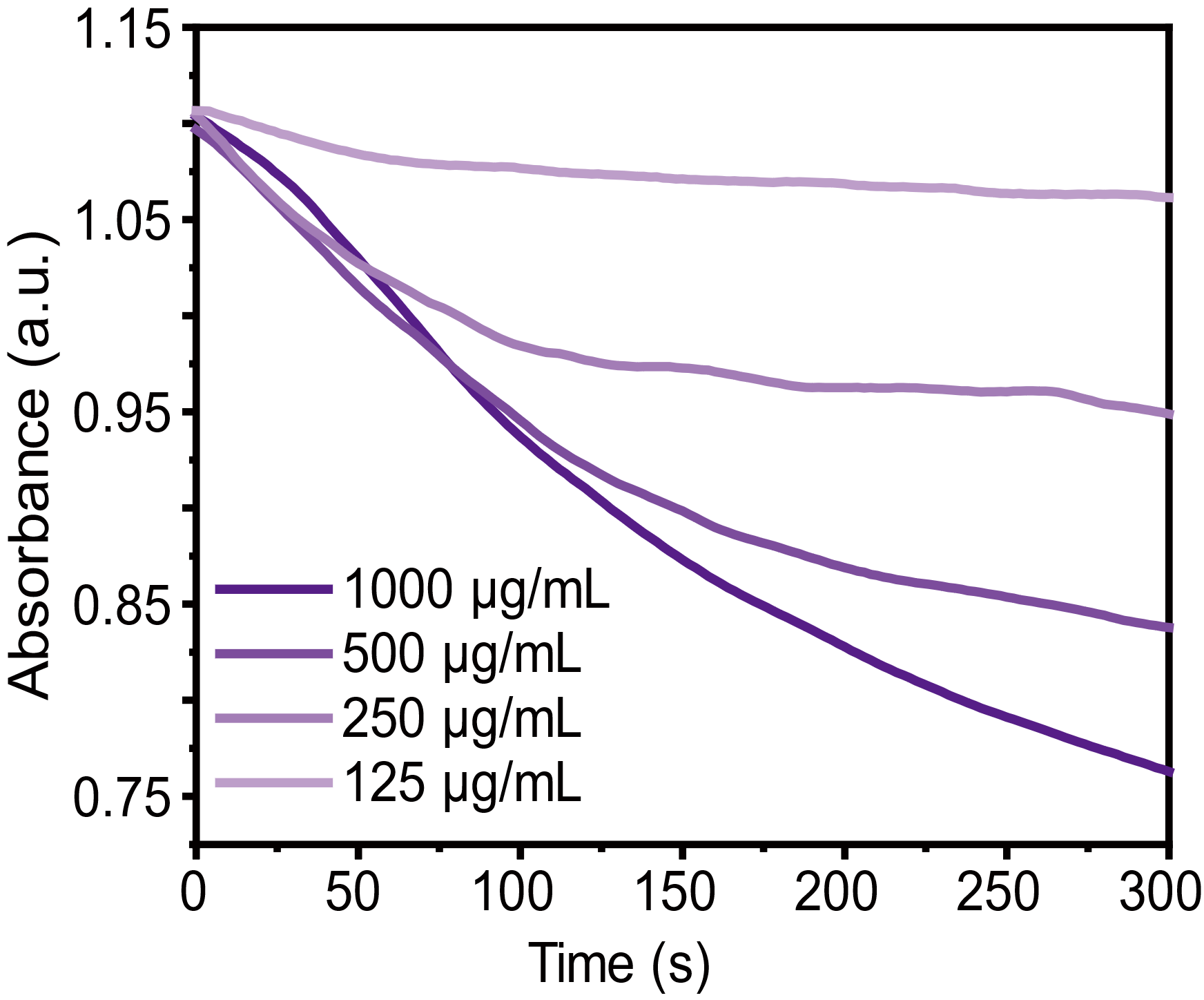


**Figure S5.** Glucose concentration and time-dependent oxidation of DPBF by GOD@POMs.


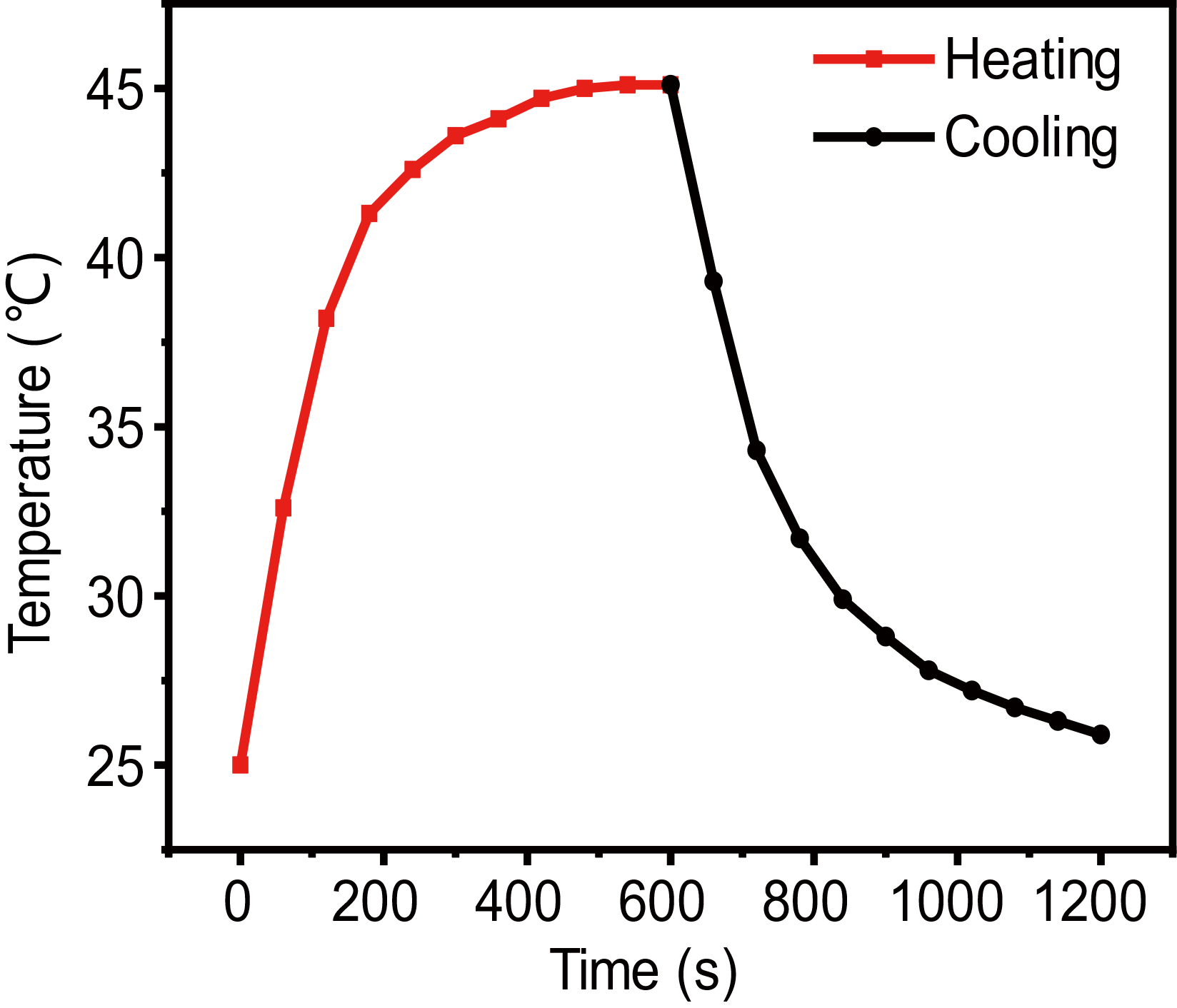


**Figure S6.** Heating and cooling curve of GOD@POMs aqueous solution under 1064 nm laser irradiation at 1.0 W/cm^2^.

The photothermal conversion efficiency (η) was calculated by following equations:

$\eta=\frac{hS(T_{\max}-T_{sur})-Q_{dis}}{I(1-10^{-A})}$ (1)

Here, *T_max_* is the equilibrium temperature and *T_sur_* is the ambient temperature. *Q_dis_* is the baseline energy generated by quartz cell and water upon laser irradiation which can be calculated independently. *I* is incident laser power. *A* is the absorbance of GOD@POMs at 1064 nm. *S* is the surface area of cell and h is heat transfer coefficient. *hS* is calculated from substituting equations:

$\theta=\frac{T-T_{sur}}{T_{\max}-T_{sur}}$ (2) *θ* is the driving force temperature.

$t=-\tau_{s}\ln\theta$ (3) *τ_s_* is the time constant in cooling period.

$hS=\frac{mc}{\tau_{s}}$ (4) Where, *m* and *c* are the mass and specific heat capacity of pure water.


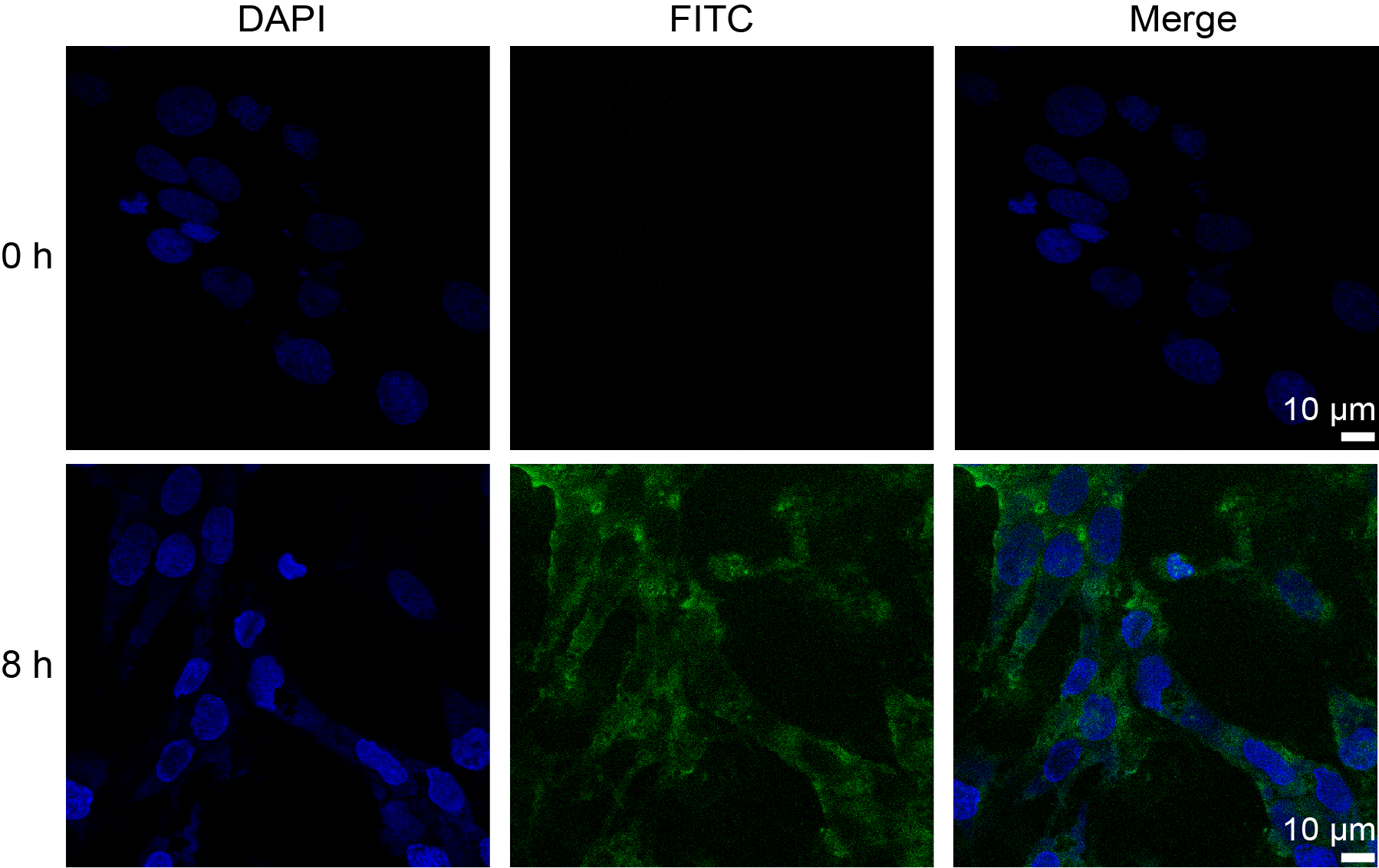


**Figure S7.** CLSM images of C6 cells incubated with FITC-labelled GOD@POMs after 8 h.


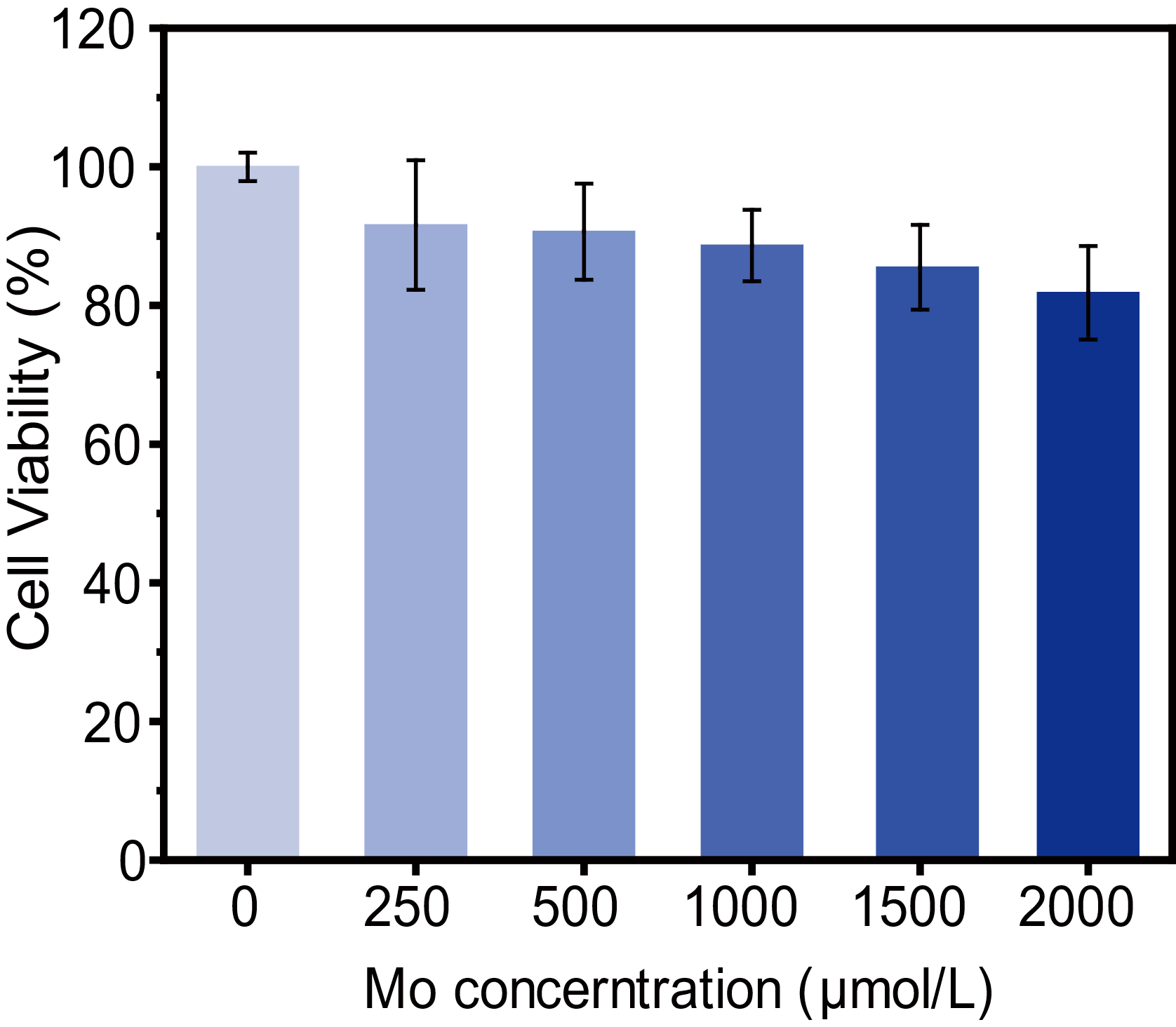


**Figure S8.** Cell viability assay of L929 cells after treatment with GOD@POMs 24 h at various concentrations.

**
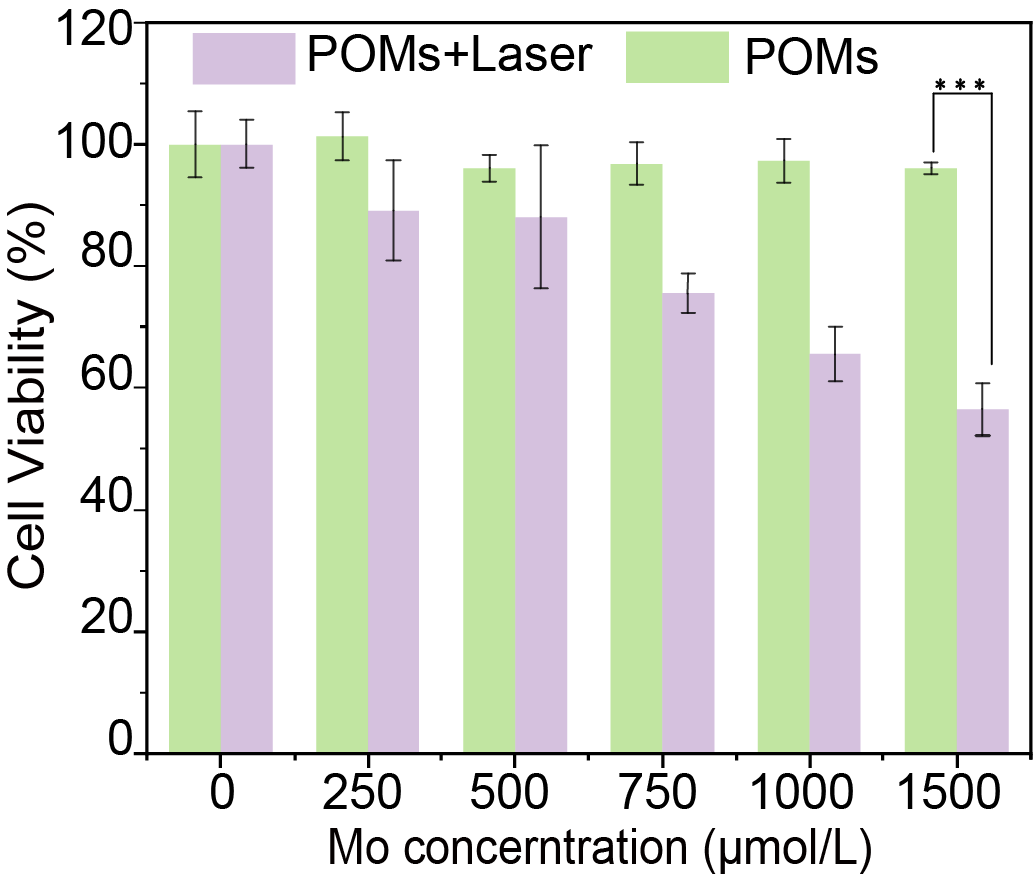
**

**Figure S9.** Cell viabilities of C6 cells after treatment with POMs at various concentrations with or without laser irradiation (1064 nm, 1 W/cm^2^, 5 min).


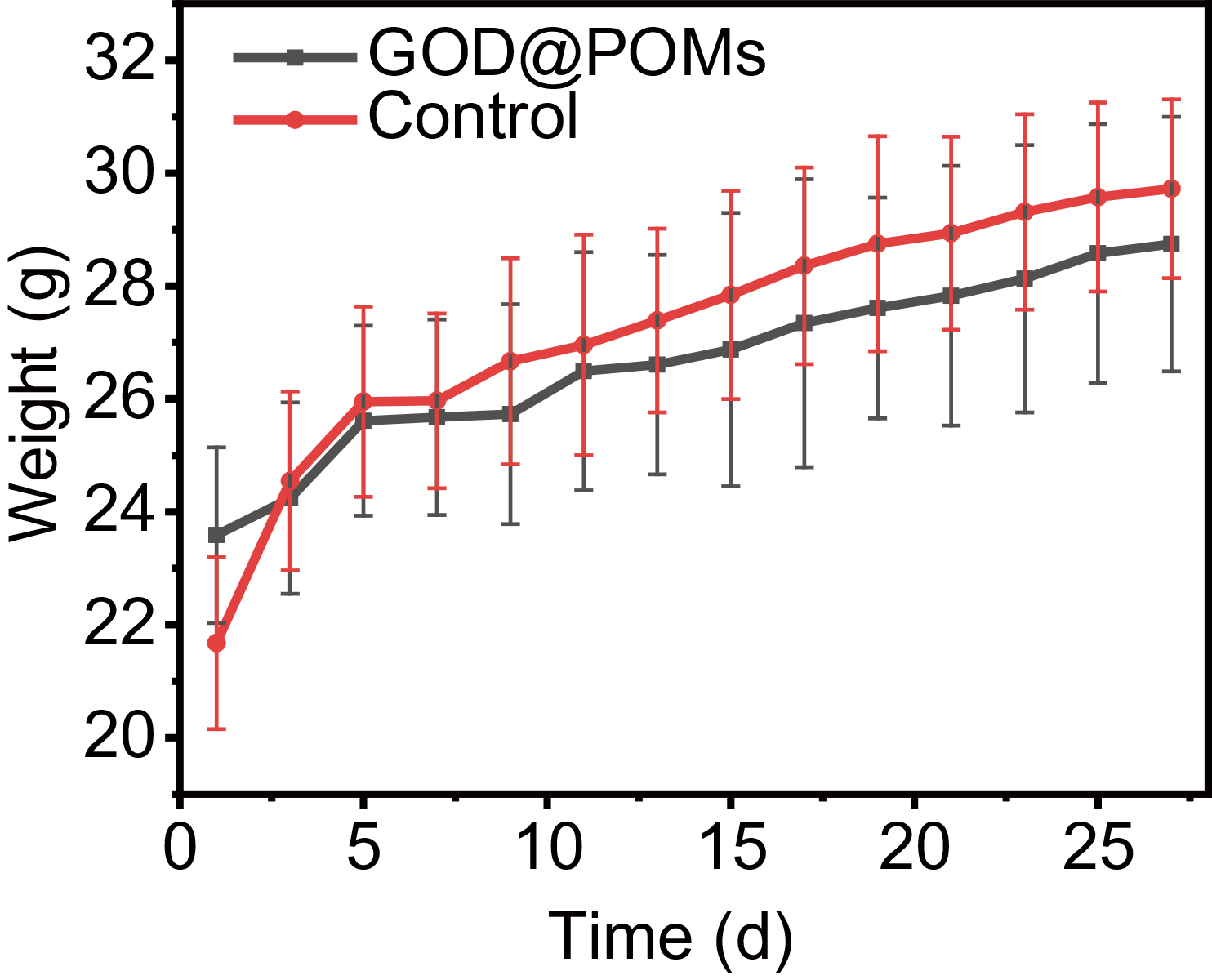


**Figure S10.** The body weight of Kunming mice during 28 days observation after different treatments.


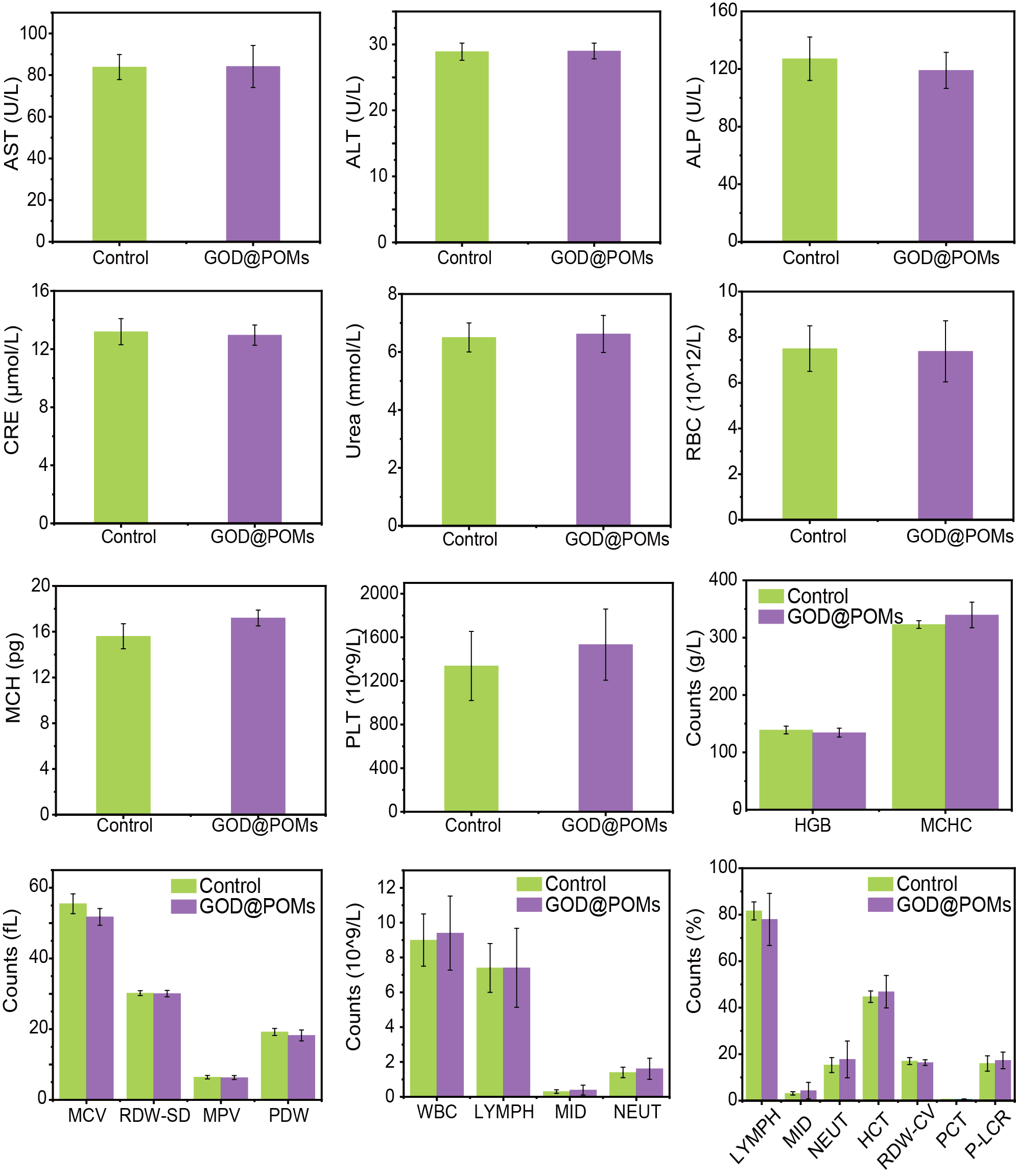


**Figure S11.** Biosafety evaluations of GOD@POMs *in vivo*. Blood biochemistry survey of liver function markers including aspartate aminotransferase (AST), alanine aminotransferase (ALT) and alkaline phosphatase (ALP). Blood biochemistry survey of kidney function markers including creatinine (CRE) and Urea. Blood analysis including red blood cells (RBC), mean hemoglobin (MCH), platelets (PLT), hemoglobin (HGB), mean hemoglobin concentration (MCHC), mean red blood cell volume (MCV), red blood cell distribution width-standard deviation (RDW-SD), mean platelet volume (MPV), platelet distribution width (PDW), white blood cells (WBC), lymphocyte (LYMPH), intermediate **cell** (MID), neutrophils (NEUT), lymphocyte (LYMPH), intermediate **cell** (MID), neutrophils (NEUT), hematocrit (HCT), red blood cell distribution width-coefficient of variation (RDW-CV), platelet hematocrit (PCT) and platelet-larger cell ratio (P-LCR) of Kunming mice after 28 days treatment.


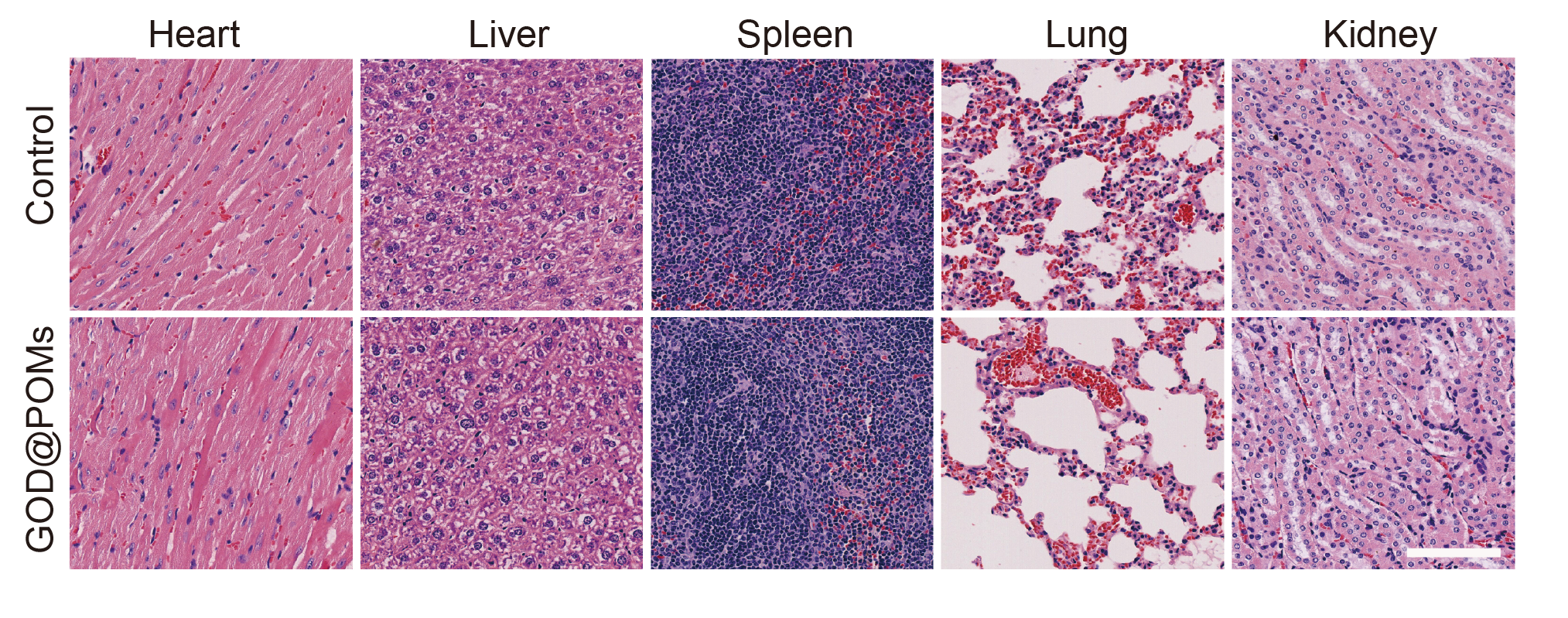


**Figure S12.** H&E staining of major organ including heart, liver, spleen, lung and kidney collected from Kunming mice after 28 days treatment. Scale bar: 100 μm.


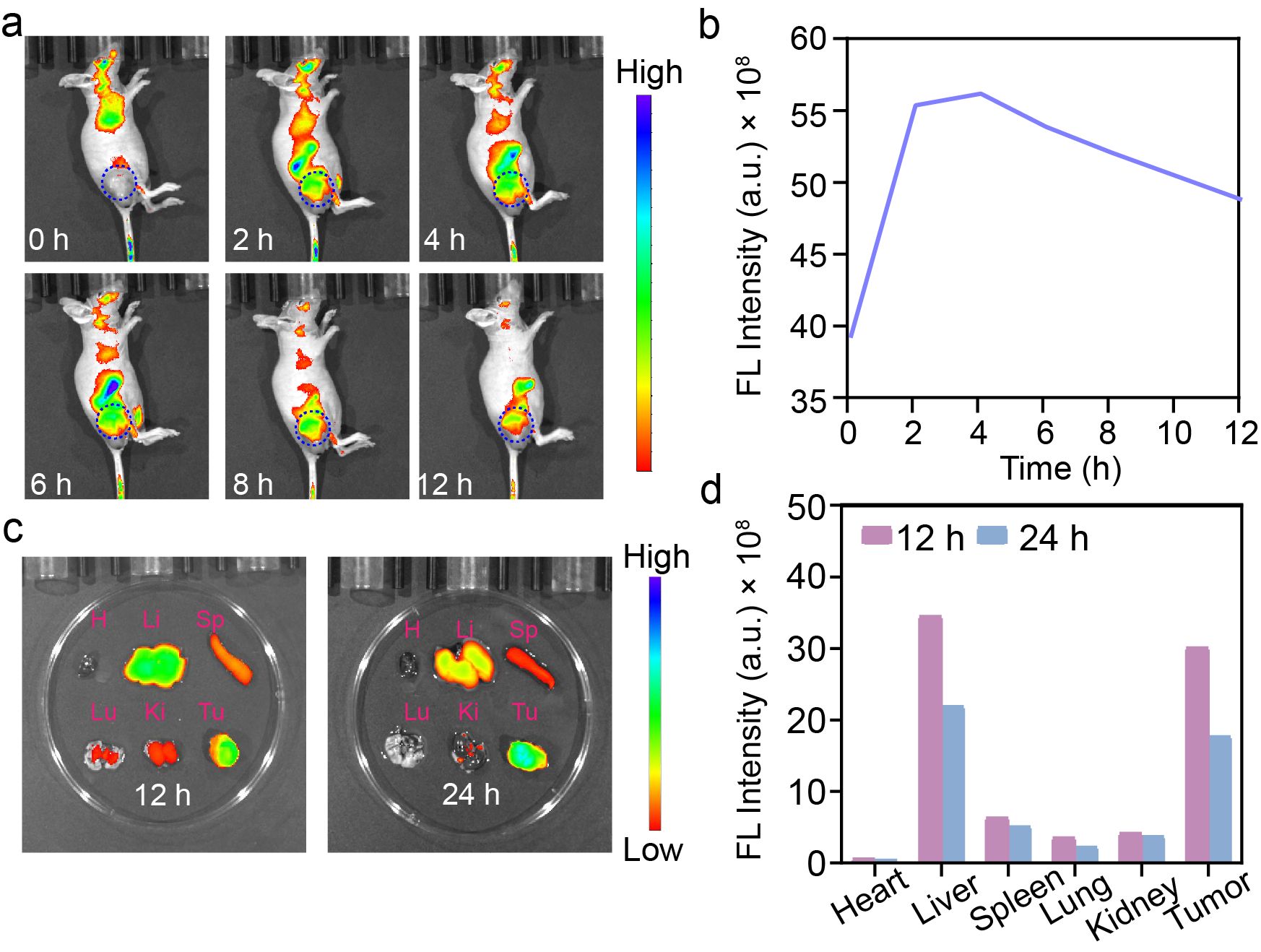


**Figure S13.** (a) Representative *in vivo* fluorescence images of C6 tumor-bearing mice at 0, 2, 4, 6, 8 and 12 h after intravenous injection of GOD@POMs. (b) Quantitative ROI assays of the fluorescence intensity at tumor site at designated time points. (c) *Ex* *vivo* fluorescence images of major organs and tumors at 12 and 24 h after intravenous injection of GOD@POMs (H: heart, Li: liver, Sp: spleen, Lu: lung, Ki: kidney, Tu: tumor). (d) Quantitative ROI assays of the *ex* *vivo* fluorescence intensity of major organs and tumors.


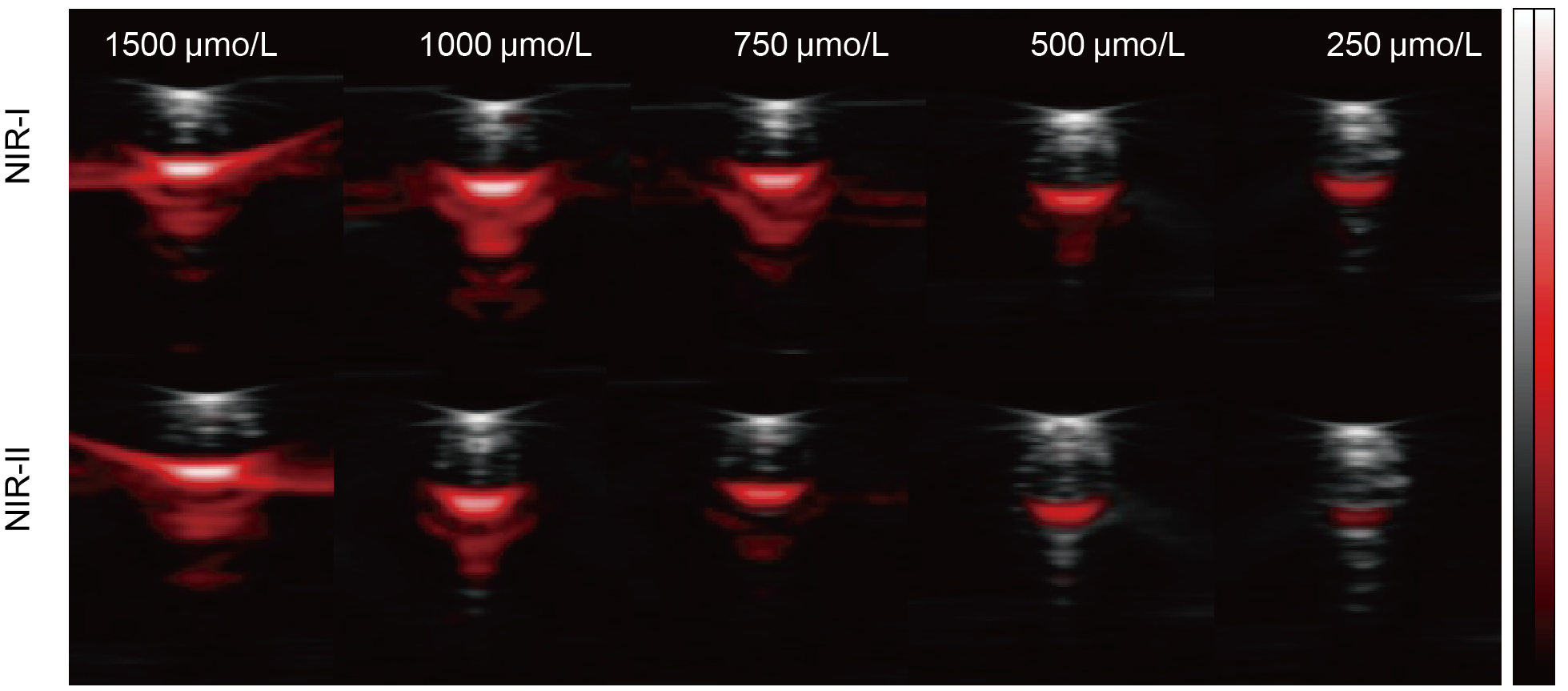


**Figure S14.** *In vitro* NIR-I and NIR-II PA images of GOD@POMs at various Mo concentration (250, 500, 750, 1000 and 1500 μmol/L).


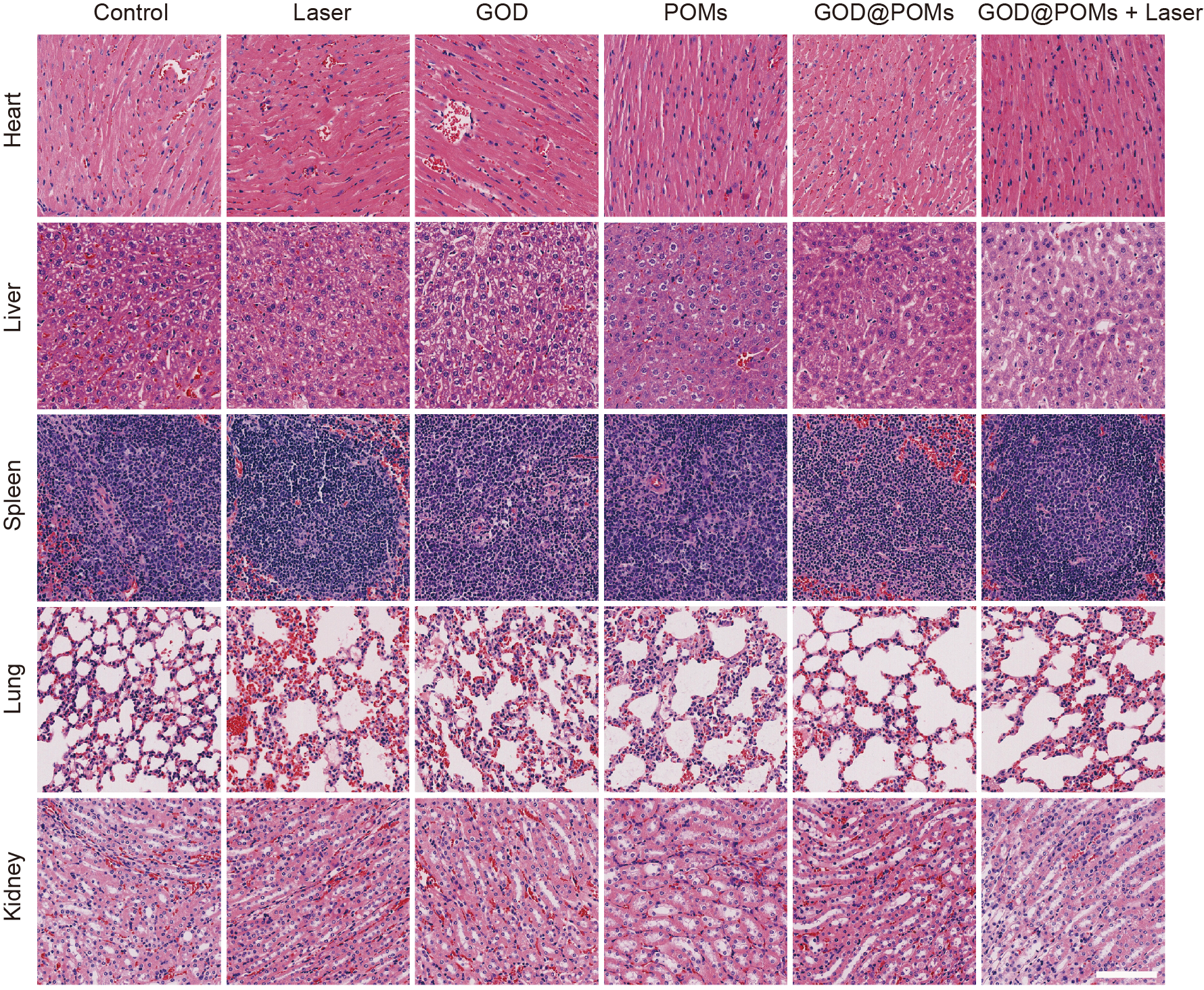


**Figure S15.** H&E staining of major organ tissues collected from mice after different treatments Scale bar: 100 μm.

**Table S1.** The light-to-heat conversion efficiency of different of photothermal agents in the reported literatures.

| Material | Wavelength (nm) | Photothermal conversion efficiency (%) | Reference |
| --- | --- | --- | --- |
| W-POM NCs | 808 | 58 | Zhou et al.  ***ACS Nano*** 14(2): 2126-2136 |
| Lip (PTQ/GA/AIPH) NPs | 1064 | 42.15 | Dai et al.  ***Small***: e2102527 |
| TiSe_2_ NSs | 808 | 65.58 | Duo et al.  ***Small*** 17(40): e2103239 |
| MnSe_2_@PVP | 808 | 39.1 | He et al.  ***Adv Mater***: e2104410 |
| PDA NPs | 808 | 27 | Yue et al.  ***ACS Nano*** 15(9): 15166-15179 |
| Micelle-Ir | 660 | 30 | Liu et al.  ***Adv Mater*** 33(32): e2100795 |
| DP-PM | 808 | 20.5 | Zhao et al.  ***Nano Lett*** 21(12): 5377-5385 |
| DDTB-DP NPs | 660 | 30.7 | Jiang et al.  ***Adv Mater*** 33(22): e2101158 |
| Pry-Ps@CP-PEG | 808 | 43.7 | Li et al.  ***Adv Mater*** 33(22): e2008481 |
| F-Pt-NPs | 808 | 28.9 | Wang et al.  ***Adv Mater*** 33(20): e2100599 |
| Pd SAzyme | 1064 | 33.98 | Chang et al.  ***Angew Chem Int Ed*** 60(23): 12971-12979 |
| GNR@SiO_2_ @MnO_2_ | 1064 | 27.47 | He et al.  ***Adv Mater*** 33(13): e2008540 |
| GOD@POMs | 1064 | 48.1 | **This work** |
